# Antimicrobial resistance in three pneumonia-causing bacterial species: the global, regional, and local state of the art

**DOI:** 10.1016/j.jgeb.2026.100802

**Published:** 2026-08-21

**Authors:** Yomna Rabie Elsherif, Dina Nadeem Abd-Elshafy, Mahmoud Mohamed Bahgat

**Affiliations:** a Department of Therapeutic Chemistry, Institute of Pharmaceutical and Drug Industries Research, the National Research Centre, Dokki, Giza 12311, Egypt; b Research Group Immune- and Biomarkers for Infection, the Centre of Excellence for Advanced Sciences, the National Research Centre, Dokki, Giza 12311, Egypt; c Environmental Virology Laboratory, Department of Water Pollution Research, Institute of Environmental Research and Climate Changes, the National Research Centre, Dokki, Giza 12311, Egypt; d Egypt Center for Research and Regenerative Medicine, Al Abbassia, Cairo 11517, Egypt

**Keywords:** Pneumonia, AMR, diagnosis, LMICs, HAI, CAP, virulence genes

## Abstract

**Background:**

Bacterial pneumonia is one of the most serious diseases worldwide. The emergence of antimicrobial resistance (AMR) magnifies its threat. Diagnosis of pneumonia is hampered by multiple causative pathogens and similar symptoms. Despite global concern, resistant strains are expand**ing with a** remarkable surveillance gap, especially in low- and middle-income countries **(**LMICs).

**Aims:**

1) Highlighting the prevalence, diagnostic approaches, and therapeutic guidelines of *Klebsiella pneumoniae*, *Pseudomonas aeruginosa*, and *Staphylococcus aureus* as key pneumonia-causing bacteria, and 2) addressing factors that impede effective surveillance.

**Methods:**

Relevant keywords were used in various databases. Results were filtered by time frame 2019-2025, followed by data selection using the Microsoft Excel application.

**Results:**

We summarized data from 161 publications, including 97 original papers, 48 reviews, 9 official websites, 6 guidelines, and 1 book chapter dealing with bacterial-hospital-, community-acquired pneumonia (HAP, CAP), and AMR. Exemplifying Egypt as a representative LMIC, the majority of isolates showed high resistance to β-lactams and aminoglycosides, while colistin was the most effective antibiotic. PCR results highlighted the high emergence of β-lactams- (ESBL) and carbapenem-resistance (CR) genes, especially the *bla*_*OXA-48*_*, bla*_*NDM*_*, bla*_*VIM*_*,* and *CTX-M15* among Egyptian isolates. The co-existence of CR with triple-, quadrable-, or more-ESBL resistance genes was reported in Egyptian *K. pneumonia isolates*. For *P. aeruginosa*, combination of both *bla*_*NDM-1*_ and *bla*_*VIM-1*_ resistance genes or ESBL and metallo-β-lactamase (MBL) genes were reported in doublet or triplet in only one Egyptian isolate. Virulence genes were detected in both *S. aureus* and *P. aeruginosa* Egyptian isolates, while only one *K. pneumoniae* was found co-harboring both AMR and virulence genes.

**Conclusions:**

1) Inappropriate use of antibiotics, maternal awareness/educational background and population's socio-cultural level play a significant role in AMR strains' spread, 2) Limited laboratory tools/infrastructure and healthcare facilities limit AMR strains' surveillance, and 3) Frequent updating national/global control plans is needed.

## 1 Background

Worldwide, pneumonia is a respiratory disease that endangers human health^1^, ^2^ due to acute lower respiratory tract infection (LRTI) by bacterial, fungal or viral pathogens.^2^, ^3^, ^4^ The disease might require long hospitalization and in the worst-case scenarios can lead to death.^5^ The infection occur through either close contact in crowded places and called community-acquired pneumonia (CAP); or after ≥ 48 hours of hospitalization and thereby named hospital-acquired pneumonia (HAP), which can happen through contaminated ventilators and thus termed ventilator-associated pneumonia (VAP).^2^, ^6^

In fact, bacterial pneumonia raises critical public health considerations, especially in LMICs.^7^, ^8^ Here, we focus on K*lebsiella pneumoniae, Pseudomonas aeruginosa, and Staphylococcus aureus* because of their notable representation in recent literature as major causative bacteria for pneumonia.

Upon presenting clinical symptoms, initially pneumonia diagnosis is confirmed by radiological or computed tomography (CT).^9^, ^10^, ^11^ The microbiological identification of the pneumonia causing bacteria is conventionally done by Gram staining and bacterial culture which are time consuming, rely on high-quality sampling, which are not easily affordable in LMICs, especially in the periphery.^12^, ^13^

Both the implication of multiple pathogens and similar clinical symptoms represent challenges for appropriate diagnosis of pneumonia^4^, ^10^ which can only be sorted out by the advanced molecular diagnostic techniques which are both faster and more specific than the traditional bacteriological methods. In addition, multiplex molecular diagnostic methods such as multiplex PCR and microarrays have been established, which are both more efficient and less time demanding than single-target assays.^14^, ^15^ Of note, the FDA approved the Unyvero- Lower Respiratory Tract-bronchoalveolar lavage, -Hospitalized Pneumonia, and BioFire FilmArray Pneumonia and Pneumonia Plus panels because of their capacity to both differentiate between several pneumonia-causing pathogens and simultaneously record antibiotic resistance genes.^12^, ^16^, ^17^, ^18^ Aided by such diagnostic tools, antibiotics could be prescribed appropriately with optimal therapeutic duration, as well as discontinuation or escalation benchmarks.^9^

Each ESBL and CR genes are highly emerged among respiratory bacterial strains in multiple localities, concomitant to their inclusion in the WHO’s detrimental pathogens list *(Supplementary Table 1).*^19^ Resistance genes are natural response that evolved due to selective pressure, antibiotic misuse, unsupervised therapy, or transferable genetic units.^20^, ^21^, ^22^, ^23^, ^24^, ^25^, ^26^ Regrettably, the AMR Collaborators internationally reviewed more than 1.2 million deaths by LRTIs related to AMR bacteria (superbugs) in 2019,^27^, ^28^, ^29^, ^30^ circa 48% (119,000) and 25% (120,000) lethality were in Africa and Europe, respectively.^13^, ^30^ However, superbugs are expected to contribute mortality rates exceeding those caused by cancer in 2050.^25^

Hence, both global and domestic strategies have been endorsed to refine infection control benchmarks, optimize antibiotic protocols, boost surveillance platforms for tracking bacterial infections and AMR strains, as well as innovate novel therapies. Notwithstanding, substantial gaps impede the successful realization of objectives globally, particularly in resource-limited countries.^31^, ^32^, ^33^, ^34^

Despite ongoing endeavors, the WHO and CDC reported mortality exceeded 740,000 among children below 5 years by pneumoniae in 2019, surpassing the fatality of any other critical infection, including HIV and malaria.^35^, ^36^, ^37^, ^38^, ^39^, ^40^ Additionally, in 2021, LRTIs were identified as one of the ten major mortality contributors worldwide.^37^ Worth noting that both etiology and incidence of the disease diverge across nations.^13^, ^30^, ^41^, ^42^ For instance, the global CAP is estimated at 1.5-14 incidence/1000 person-years,^10^, ^43^ while the incidence density of VAP is reviewed as varying at 3.6, 7.9, 8.1, and 8.8/1000 ventilator-days in Saudi Arabia, Iran, Lebanon, and Kuwait, respectively.^41^ Meanwhile, LRTIs’ mortality/annum surpassed 1000/100,000 population in the Middle East and North Africa,^40^ the WHO ranked them the ninth cause of death in Egypt by 17.7 deaths/100,000 population in 2021.^44^ Moreover, it was shown that HAP occupied nearly 65% of total Egyptian hospital-acquired infections (HAIs) in various districts.^45^

Therefore, here we are going to outline the global pattern of the three principal bacteria for pneumonia, exemplifying Egypt as a LMIC. Specifically, we sought to highlight the following: 1) the prevalence of bacterial pneumonia in both HAIs and CAP; 2) different diagnostic tools for bacterial pneumonia and AMR identification; 3) factors that elevate bacterial pneumonia burdens in LMICs; and 4) global and national initiatives for AMR control, to identify existing gaps and inform targeted interventions.

## 2 Methods:

**Searching criteria:** We conducted a structured narrative review using various databases, including PubMed, Google Scholar, WHO, CDC, and the Global Burden of Disease on bacterial pneumonia in HAI and CAP settings at global, regional, and local levels. Because of the exuberance of data for 3 key pneumonia causing bacteria (*K. pneumoniae, P. aeruginosa,* and *S. aureus*), we continue our search process focusing on them in a combination with keywords such as "pneumonia prevalence," "antimicrobial resistance," "pneumonia surveillance," "ventilator-associated pneumonia," "molecular detection," "MDR pneumonia epidemiology," "virulence factors," "infection control policy," "global action plan," and "antibiotic stewardship". We restricted the search process to publications from 2019 to 2025.

**Inclusion criteria:** peer-reviewed research articles (original and reviews), official website data (global and national health authorities), surveillance datasets and reports, and policy documents which address *K. pneumoniae, P. aeruginosa,* and *S. aureus* pneumonia epidemiology, detection methods, AMR, and infection-control policy in HAI/CAP settings dealing with, with particular relevance to global, regional, and local studies, focusing on LMIC context.

**Exclusion criteria:** non-peer-reviewed commentaries/editorials, studies not addressing bacterial pneumonia, and articles published before 2019 unless of established historical/methodological relevance.

**Screening and analysis process:** titles and abstracts were manually screened against these criteria for initial selection. Then, full texts of shortlisted articles were independently assessed for eligibility. Data extracted included study location, methodologies, isolate type, diagnostic techniques, pathogen, antimicrobial susceptibilities, AMR and virulence profiles based on both phenotypic and phylogenetic assays, and policy relevance. We tabulated the data in Excel sheets.

## 3 Results

**Figure 1:**
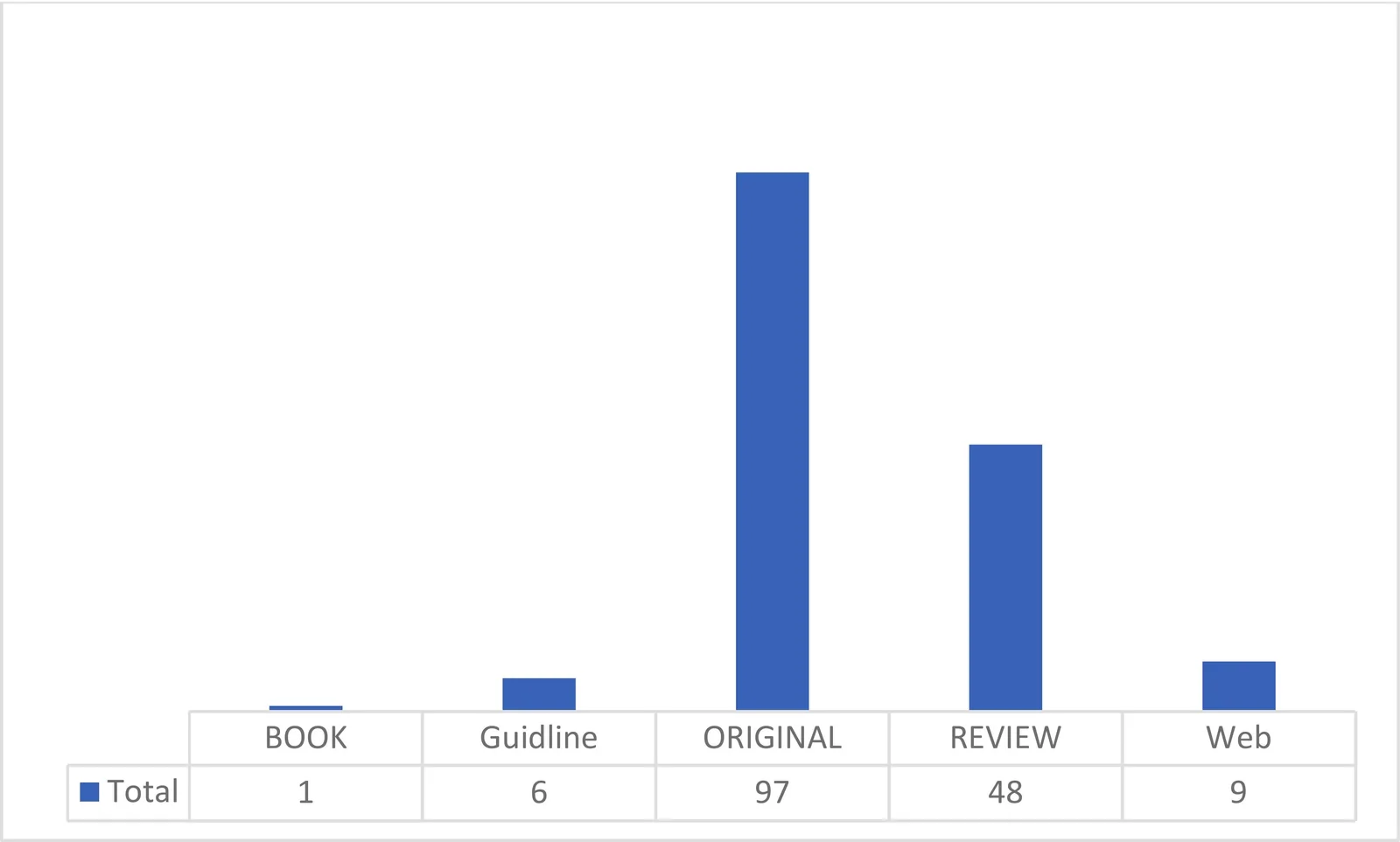
Types of the reviewed literature.

**Figure 2:**
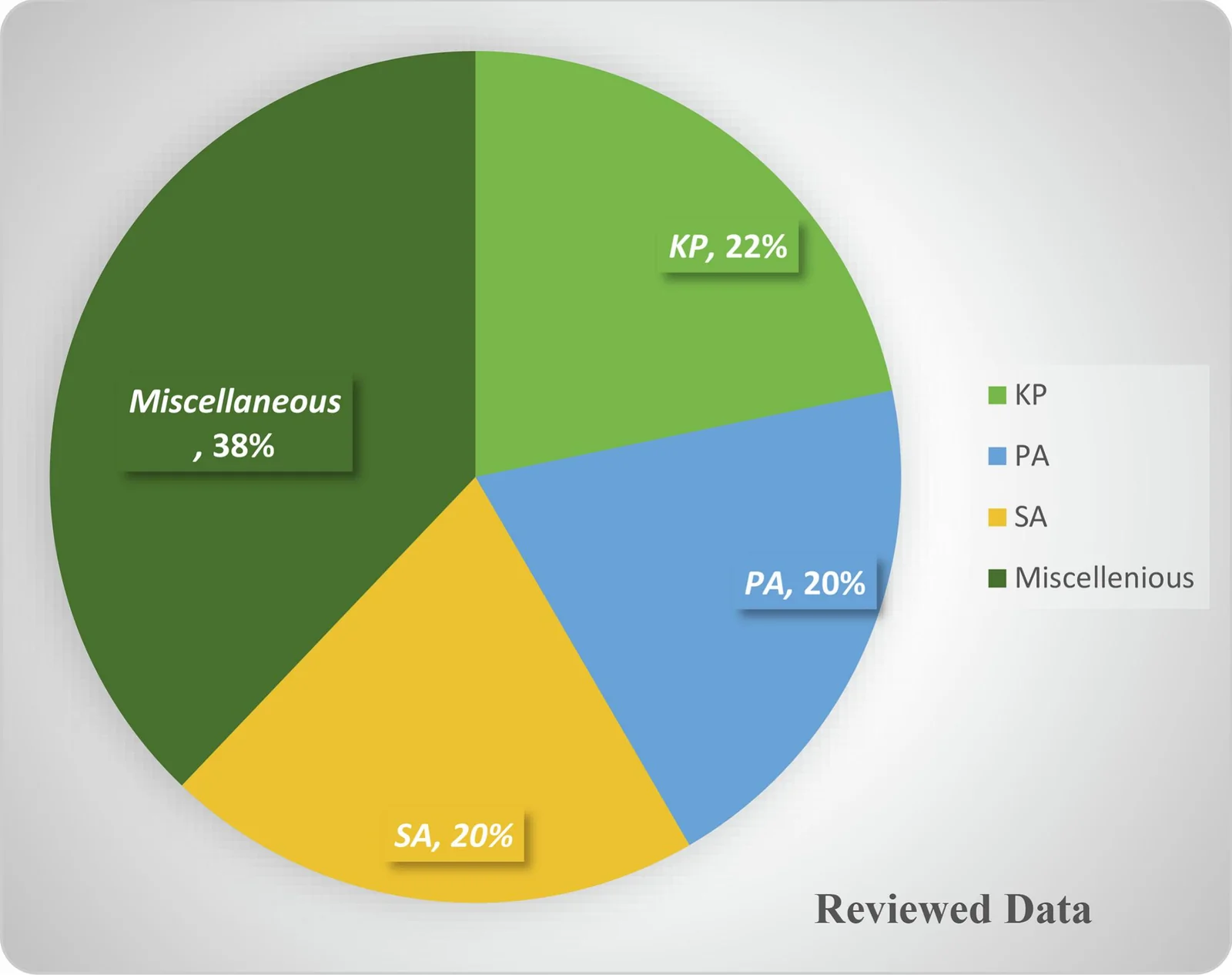
Data distribution in the reviewed literature. *KP = Klebsiella pneumoniae*; *PA= Pseudomonas aeruginosa*; *SA =Staphylococcus aureus*; Miscellaneous= data of mixed bacterial species and themes, such as HAI, AMR, CAP, regulations, and guidelines.

The research process resulted in 161 articles. Of these, 79 were original studies, 48 were review articles including 9 systematic and meta-analyses reviews, 9 official websites, 6 guidelines, and a book chapter *(*Figure 1*)*, all were written in English, unless only one Arabic guideline. Entire manuscripts were reviewed, except 1 review article and 8 original studies were only available as abstracts. Furthermore, we classified the reviewed literature according to the pathogens: *K. pneumoniae (n= 35), S. aureus (n= 33), and P. aeruginosa (n= 32). Meanwhile,* the rest of articles (miscellaneous, n= 61) informed data of mixed bacterial species and themes, like HAI, AMR, CAP, regulations, and guidelines *(*Figure 2*).*

## 4 Pneumonia-causing bacteria:

### 4.1 Klebsiella pneumoniae (K. pneumoniae)

#### 4.1.1 Classification and pathogenesis

It is a Gram-negative bacillus member of *Enterobacteriaceae,*^26^, ^46^ and it is frequently reported to cause both CAI and HAI nationally and globally,^46^, ^47^, ^48^ particularly in the intensive care units (ICUs) of neonates and adults.^49^, ^50^ Besides pneumonia, it can lead to meningitis, cystitis, endotoxic shock, lung abscesses, septicemia, endocarditis, or other infections of the urinary tract, surgical wound, skin and soft-tissue, and otitis media.^47^, ^51^

#### 4.1.2 Diagnosis

Various techniques were applied to detect *K. pneumoniae* and distinguish it from other *Enterobacteriaceae* species, like Voges-Proskauer, Gram staining, and citrate reaction.^51^ Notably, Carl Friedländer early regarded *K. pneumoniae* as a pneumonia-causing pathogen in 1882,^52^ upon staining the fibrinous discharge of bronchial tissue samples from pneumonia cases with aniline dyes through the Weigert-Koch approach.^53^

#### 4.1.3 Therapy

An empirical therapy of broad-spectrum antibiotics is often administered in CAP and HAP, including macrolides (e.g., azithromycin), β-lactam/β-lactamase inhibitor (e.g., piperacillin-tazobactam or amoxicillin/clavulanate), or third-generation cephalosporins.^54^, ^55^ Although AMR originates from spontaneous mutations in encoded genes or horizontal gene transfer, the selective pressure exerted by antibiotic use accelerates emergence and spread of antibiotic-resistant, multidrug-resistant (MDR), as well as extensively drug-resistant (XDR) strains.^56^ Consequently, carbapenem antibiotics are listed as the last option and a life-saving intervention for such critical infections.^26^ Carbapenems (i.e., imipenem, meropenem, doripenem, and ertapenem) are the latest β-lactam antibiotic members having sulfur-free and unsaturated ring.^57^, ^58^, ^59^ This unique structure shielded them from lysis by most β-lactamases and makes them “the last resort” to counter infections caused by pathogens tolerating almost all antibiotics.^57^, ^58^, ^59^ However, carbapenem resistance genes were expeditiously evolved.^31^ In 1996, the first recognized carbapenemase-expressing *K. pneumoniae* was in isolates from the USA, then it broadened to other localities in the world, including Italy, Colombia, Brazil, China, and others.^60^, ^61^, ^62^

These resistance genes could be encoded in plasmids and having different mechanisms, such as: a) expression of destructive enzymes named β-lactamases, hydrolyzing the majority of β-lactam antibiotics, including carbapenems, and b) excessive expression of efflux pumps to expel antibiotics and inhibit their presence in the periplasm through alteration of the cell wall permeability.^60^, ^63^, ^64^

Remarkably, the WHO placed CR *K. pneumoniae* starins in the first rank of the bacterial priority list by 2024 after being the sixth in 2017; while the third-generation cephalosporin-resistant strains were inserted in 2024 in the sixth position after nonexistence in the prior version,^65^ which could manifest their threat *(Supplementary Table 1).*^19^

Therefore, efforts were exerted to detect carbapenemases either through phenotypic or genotypic assays. The genotypic approach is distinguished by its ability to clarify the CR mechanism, rather than the phenotypic methods which just recognize the production of carbapenemases.^66^ Accordingly, carbapenemases are clustered into two major categories: *MBLs* (class B), which are zinc-dependent, and non-metallo-carbapenemases (classes A, C, as well as D), which are zinc-independent. Carbapenemases class B (Verona integron-encoded metallo-β-lactamase (*VIM)*, imipenemase (*IMP*), New Delhi metallo-β-lactamase (*NDM)*, and Guiana extended-spectrum (*GES*)), class D (oxacillinase-48 (*OXA*-*48*)), and class A *K. pneumoniae* carbapenemase (*KPC*) are frequently involved genes in the molecular detection of CR-*KP.*^14^, ^26^, ^31^, ^48^, ^67^, ^68^ The *OXA*-*48* and *NDM* are the top genes of concern within different published research studies for CR *K. pneumoniae* isolates.

Indicatively, colistin is used as a last choice for treatment of CR bacterial infection in co-administration with other antibiotics to minimize its cytotoxicity and hetero-resistance that are observed upon being utilized singly.^69^

#### 4.1.4 Prevalence

##### 4.1.4.1 Globally

Worldwide prevalence of *K. pneumoniae*, especially AMR strains was reviewed by the European antimicrobial resistance collaborators in a systematic analysis of many reports. In Europe in 2019, the AMR *K. pn*eumoniae was associated with 68,994 mortalities, and was classified by the WHO European region to be resistant for aminoglycosides, β-lactam/β-lactamase inhibitor, carbapenems, fluoroquinolones, third-generation cephalosporins, and trimethoprim-sulfamethoxazole, that later was the cause of 34900, 60600, 23100, 47100, 52300, and 50400 mortalities, respectively. In Russia, which according to the WHO European region has the highest population, AMR *K. pn*eumoniae contributed to death of more than 5000 humans. Uzbekistan observed the highest mortality rate (13.6 deaths/100,000) associated with AMR *K. pneumoniae*, and Switzerland showed the lowest mortality rate (0.44/100,000).^30^

The first reported outbreak observed for *K. pneumoniae producing GES-5* and *SHV*-*12 ESBL* genes in Korea was in 2005. The *GES-5* and *SHV*-*12* genes were detected during this outbreak by PCR on sputum, pus, urine, and bile isolates.^70^ In Turkey, the first reported massive nosocomial outbreak of *K. pneumoniae* expressing the *OXA-48* gene was in 2008 and the detection was done by conventional PCR on blood culture, endotracheal aspirate, catheter, wound, pus, ascites, and urine isolates where many *OXA-48-*producing *K. pneumoniae* coexisted with other *ESBL* genes such as *SHV-12* or *CTX-M-15.*^71^ Later, *OXA*-*48*-producing *K. pneumoniae* was considered endemic in Turkey.^72^

In Sweden, *K. pneumonia*e *NDM*-*1* CR gene was reported in a urine from a Swedish-Indian patient who traveled to New Delhi followed by its expansion into other countries including the United Kingdom, the United States, Australia, Germany, Greece, Span, Canada, India, Italy, France, Belgium, and Kenya.^71^, ^73^

##### 4.1.4.2 Regionally

In Saudi Arabia, *K. pneumoniae* causes the most CAP infections.^42^ Additionally, third-generation cephalosporin-resistant *K. pneumoniae* strains associated with 53% of deaths due to AMR across Africa.^13^

Meanwhile, Oman had reported the first *NDM-1*-positive *K. pneumoniae* in the Middle East and the Arabian region in 2010^65^ which was identified by the API20E manual biochemical test, and their antibiotic susceptibility was tested through the disk diffusion method, followed by PCR and sequencing for β-lactamase genes in both class A and B using primers based on foreign references.^74^ When an Iraqi soldier was wounded in his shoulder during the terrorist attack at the Baghdad cathedral in October 2010, he was initially hospitalized in Baghdad before being admitted to the Bicêtre hospital in Paris, his PCR, sequencing, and plasmid results indicated his infection with both *bla*_NDM-1_ carbapenamase and *bla*_CTX-M-15_ ESBL-*K. pneumoniae.*^75^

Within the same period, Tunisia reported *OXA-48* for the first time,^76^ followed by the first report of ST15 strain carrying the *OXA-48* gene using the BIORAD kit on sputum, urine, throat, and stool isolates of hospitalized patients.^77^ Later, the first WGS of *NDM-1-*positive *K. pneumonia* from clinical isolates was recorded at the Tunisian university hospital in 2018.^78^ Of note*,* both the *K. pneumonia NDM* and *OXA-48* are the most recorded CR genes in Tunisian patients.^77^, ^78^

In 2017, an Algerian patient hospitalized in Annaba University Hospital was the first reported case infected with two strains of CR *K. pneumoniae* harboring the *NDM-1* gene using PCR-specific primers from previously published references.^79^ Moreover, in 2019, Algerian researchers designed 100% specific primers for the mcr-8 gene that causes colistin resistance based on published sequences of mcr gene variants on the GenBank then used these in both RT-PCR and sequencing on clinical bacterial isolates. Both the sensitivity and specificity of the designed PCR primers were confirmed by matrix-assisted laser desorption ionization-time of flight mass spectrometry (MALDI-TOF-MS).^80^

##### 4.1.4.3 Locally

An Egyptian team reviewed the existence of *K. pneumoniae* in Tanta, Zagazig, Assiut, Cairo, Giza, Ismailia, and other Egyptian localities, where the recorded prevalence of CR and ESBL-producing *K. pneumoniae* strains in Egyptian hospitals from 2004 to 2020 showed significantly broad ranges, which reflects a gap in the AMR monitoring system.^45^

**Figure 3:**
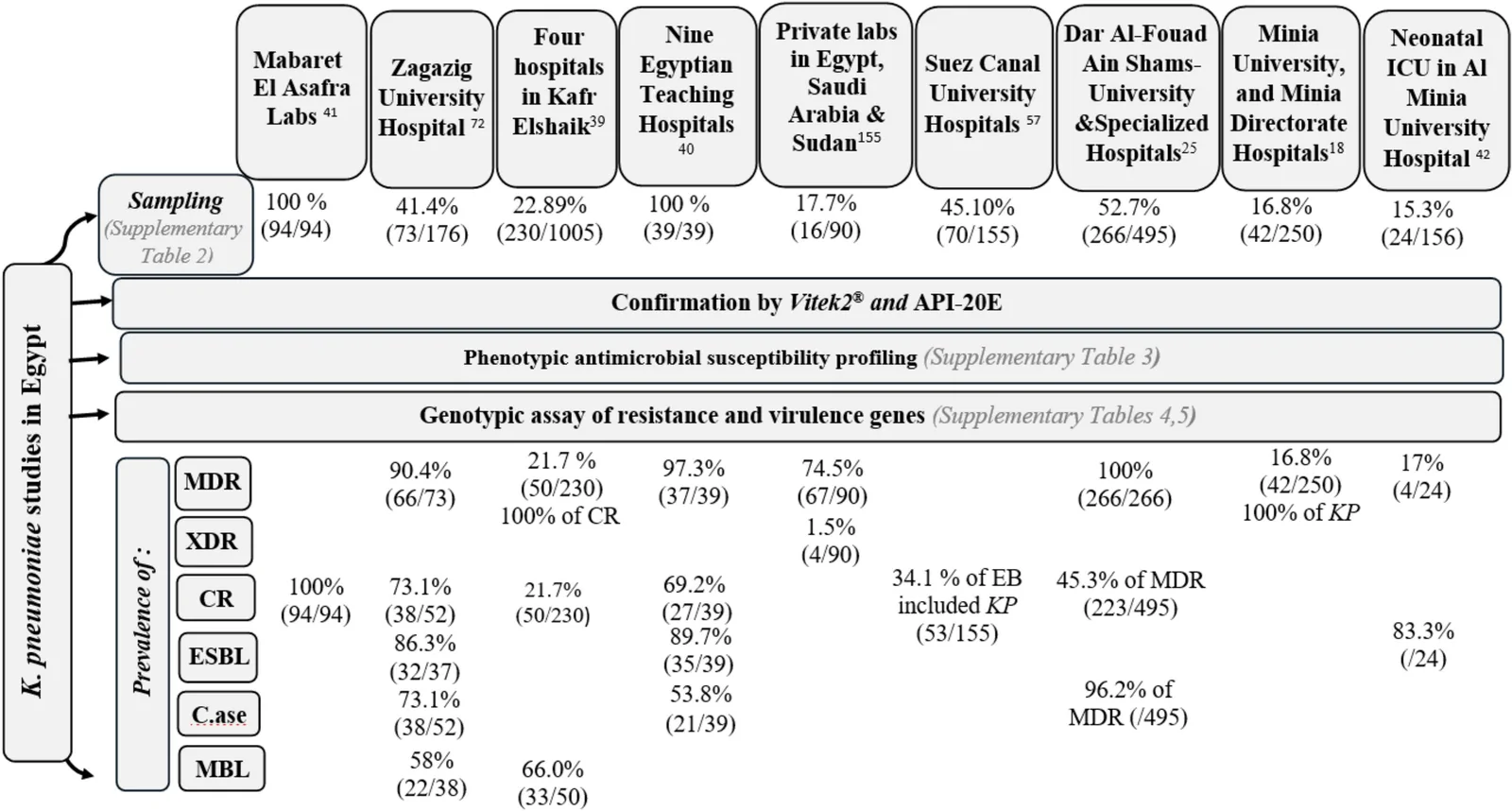
Flowchart of recent *Klebsiella pneumoniae* investigations in Egyptian studies. Nein collaborating Egyptian teaching hospitals: National Cancer Institute (NCI), Cairo, Alexandria University Research Center, Greater Cairo Tertiary Hospital, and Outpatient Laboratory; Assiut-, Banha-, Mansoura-, Benisweef-, Fayoum-, and Sohag- University Hospitals.; KP= Klebsiella pneumoniae; MDR= multi-drug resistant; XDR= extended-drug resistant; ESBL= extended spectrum β lactam; Case= carpabenamase producing strains; MBL= metalo- β lactamase producers; EB= Enterobacteriaceae.

Recently, various studies were conducted in Egypt in several governorates including Cairo, Alexandria, Suez, Kafr Elshaikh, Benisweef, Fayoum, and others on a small research scale to determine *K. pneumonia*e prevalence, the emergence of MDR and XDR strains, susceptibility to different antibacterial classes, the existence of AMR and virulence genes and we summarized their results in Figure 3 and *Supplementary Table 2.*

The identification was done by classical bacteriological and biological methods including colonial morphology, motility indole ornithine, Gram-stained films, tube indole, citrate, urease, lysine iron agar, methyl red Voges Proskauer, triple sugar iron, and carbohydrate fermentation tests followed by the Vitek2® automated system and the API-20E manual panel to confirm the results.^24^, ^26^, ^64^, ^69^

In these studies, β-lactams, β-lactam/β-lactamase inhibitor combination, carbapenems, and other therapeutic antibacterial classes revealed medium to high resistance *(Supplementary Table 3)*. Of the carbapenem antibiotics, meropenem showed high resistance rates in Alexandria, Minia (neonatal ICU), Zagazig which were 100% (94/94), 95.8% (23/24), and 71.2% (52/73), respectively.^48^, ^49^, ^69^ Unlikely, Colistin showed the best susceptibility rates which were 72.2% (/94), 89% (/73), and 92.7% (/266) in Alexandria, Zagazig, and Cairo, respectively *(Supplementary Table 3).*^48^, ^69^

Many molecular studies investigated presence of both the CR and ESBL genes in *K. pneumoniae* isolates relying mostly on previously published primers, imported multiplex PCR or microarray kits *(Supplementary Table 4)*. These studies were mainly concerned with the *bla*_*OXA-48*_*, bla*_*NDM*_*, bla*_*VIM*_, and *bla*_*KPC*_ CR genes, however, other CR genes harbored in *Klebsiella* isolates, such *as bla*_*IMI*_*, bla*_*OXA181*_*, bla*_*OXA-232*_*,* and *bla*_*OXA-244*_ were recorded in minor percentages.

In 2020, a study in Egypt reported *K. pneumoniae* with a prevalence of 15.30% (24/ 156) in blood isolates from the neonatal ICU in Minia University Hospital, of which 17% (/24) and 83.3% (/24) were MDR and XDR, respectively.^81^ Other study from both Minia University and Minia Directorate Hospitals recorded 16.8% (42/250) MDR *K*. *pneumoniae*in isolates in urine, wound swabs, sputum, ICU, and orthopedic wards at prevalence rates of 18.1% (16/88), 17.3% (13/75), 14.9% (13/87), 41.9% (13/31), 6.25% (2/32), respectively *(Supplementary Table 2).*^24^

Another report pneumonia in Ain Shams University-, Ain Shams Specialized University-, and Dar Al-Fouad-Hospitals in Cairo detected 52.7% (266/495) MDR *K. pneumonia* in intra-abdominal, urinary tract, and lower respiratory tract infections at prevalence rates 52.2% (91/181), 43% (80/186), 71.1% (91/128), respectively.^31^ Using the Netherlands microarray kit, results also showed the detection of the ESBL resistance genes including the *bla*_*CTX-M15*_ and *bla*_*SHV-OSBL*_ across isolates at levels of 71.4% (190/266) and 51.1% (136/266), respectively.^31^ Furthermore, they detected minor percentages of the CR genes *(Supplementary Table 4)*, and recorded their co-existence with the ESBL resistance genes (three genes, such as *bla*_*SHV-OSBL*_*, bla*_*CTX-M-15*_*,* and *bla*_*NDM-1*_; four genes, such as b*la*_*SHV-OSBL*_*, bla*_*TEM-OSBL*_*, bla*_*CTX-M-15*_*,* and *bla*_*NDM-1*_; and more than four genes, such as *bla*_*SHV-OSBL*_*, bla*_*TEM-OSBL*_*, bla*_*CTX-M-15*_*, bla*_*CTX-M-14*_*, bla*_*NDM-1*_*,* and *bla*_*OXA-48*_*).*^31^

A study conducted in 2021 in the ICU of a tertiary referral hospital servicing five governorates in eastern Egypt, the Zagazig University Hospital, recorded 41.40% (73/176*) K. pneumoniae* among endotracheal aspirates of HAP patients which included 90.4% (66/73) MDR, 86.3% (/37) ESBL, 73.1% (38/52) CR. Phenotyping assay classified the CR group into 73.1% (38/52) carbapenemase- and 58% (22/38) MBL-producers *(Supplementary Table 2)*; while genotyping by two multiplex PCR kits detected 50% (26/52) *bla*_*NDM*_ and 36.5% (19/52) *bla*_*OXA-48*_. Moreover, co-harboring of two resistance genes namely *bla*_*OXA-48*_ and *bla*_*NDM*_ was seen in nine isolates (17.3% (9/52)) *(Supplementary Table 4).*^69^

WGS on thirty-nine MDR *K. pneumoniae* isolated from nine different Egyptian hospitals in Cairo, Alexandria, Assiut, Banha, Mansoura, Benisweef, Fayoum, and Sohag from blood culture, respiratory samples, wound, and UTI isolates in 43.6% (n= 17), 25.6% (n= 10), 15.4% (n= 6), and 15.4% (n= 6), respectively *(Supplementary Table 2)* revealed presence of the Yersiniabactin, Aerobactin, and Hyper-Mucoviscosity virulence genes in 69.2% (n= 27), 41% (n= 16), and 15.4% (n= 6) of the MDR *K. pneumonia*e isolates, respectively *(Supplementary Table 5)* and recorded the first co-existence of AMR and virulence genes in single strains in Egypt.^47^ In addition, results identified *K. quasipneumoniae* subsp*. similipneumoniae* in blood culture in two cases.^47^ In 2024, four multiplex PCR kits where used in a study conducted in Alexandria to investigate the presence of eleven virulence genes among 94 CR *K. pneumoniae isolates* divided into 54.3% (n= 51) blood, 13.8% (n= 13) wound swab, 8.5% (n= 8) tracheal aspirate, 8.5% (n= 8) sputum, 8.5% (n= 8) urine, 5.3% (n= 5) bronchoalveolar lavage, and 1.1% (n= 1) catheter *(Supplementary Table 2).*^48^ Results showed presence of the enterobactin siderophore system (EntB); outer membrane lipoprotein (ycfM), type 3 fimbrial adhesion (mrkD), type 1 fimbriae (fimH), yersiniabactin siderophore system (ybtS), uridine diphosphate galacturonate 4-epimeras (uge), aerobactin siderophore system (iutA), the regulator of mucoid phenotype A (rmpA), like fimbrial adhesion (Kpn); while both the mucoviscosity-associated gene A (*magA)* and K2 capsule serotype *(k2A)* were absent in all isolates *(Supplementary Table 5).*^48^

### 4.2 Pseudomonas aeruginosa (P. aeruginosa)

#### 4.2.1 Classification and pathogenesis

*P*. *aeruginosa* is a Gram-negative rod-shaped bacterium. Worldwide, it is one of the most critical nosocomial infection-causing bacteria. It could cause a broad range of diseases including pneumonia, UTIs, wound and burn infections, endocarditis, sepsis, and bacteremia.^23^

It had been discovered in 1882 by the French pharmacist Carle Gessard, who observed its ability to produce a bluish-green color in culture and thus named it *aeruginosa* which means “bluish green” both in the Italian and Greek cultures. This color is due to production of pyocyanin which is a phenazine blue/green compound of both antimicrobial and toxin effects.^82^

#### 4.2.2 Diagnosis

Using conventional methods based on their biological characteristics, including oxidase, arginine hydrolase, acetamidase tests, and pyocyanin production.^83^ However, efforts were spent to describe more specific and sensitive detection methods. In 1945, Harper and Cawston described a modified culture media to improve *P. aeruginosa* growth, followed by Lowbury and colleagues who showed that supplementing growth medium with 0.03% cetrimide selectively promotes growth of *P. aeruginosa.*^83^, ^84^, ^85^, ^86^ Later Brown and his group developed the Lemco-based selective medium which boss pyocyanin secretion.^83^, ^87^

#### 4.2.3 Therapy

Since *P. aeruginosa* developed resistance against various β-lactam antibiotics, carbapenem were prescribed as the antibiotics of choice to preserve life upon infection with the ESBL strains.^88^ Consequently, MBLs in *P. aeruginosa* strains were recorded in various outbreaks in many regions of the world, including Korea, Kenya, Canada, Greece, and Italy.^89^, ^90^, ^91^, ^92^ Nevertheless, the extensive use of carbapenem antibiotics caused emergence of CR *P.* a*eruginosa* strains*.* The WHO listed in 2024, the CR *P. aeruginosa* as the 10^th^ serious resistant bacteria after being the 2^nd^ in 2014 *(Supplementary Table 1).*^19^

Of note, the first reported *P. aeruginosa* isolates harboring the NDM CR gene were in 2011 in Serbia.^93^

#### 4.2.4 Prevalence

##### 4.2.4.1 Globally

The literature reflected a significant advanced reporting of MDR *P. aeruginosa* strains in blood, sputum, bronchial fluids, BAL, and other specimens of hospitalized patients in various regions, including China, Canada, Germany, and the USA.^94^, ^95^, ^96^, ^97^

In 2021, Wang *et al.* extracted 2953 NCBI-uploaded human-derived *P. aeruginosa* genomes from 40 countries in Asia, Europe, the Americas, Africa, and Australia. Sequence analysis showed that 11.1% (328/2953) of the sequences carried the CR genes with prevalence of 50.3% (165/328) bla_VIM_, 39.3% (129/328) bla_IMP_, 7.9% (26/328) bla_NDM_, and 3.0% (10/328) bla_KPC_. Of note, absence of the bla_OXA-48-like_ gene was recorded, while co-harboring of both bla_VIM_ and bla_IMP_ was only seen in two isolates. Last, this work showed the variation in the prevalence of the the emerged CR *P. aeruginosa* strains among countries which was 50.45% (112/222), 23.08% (9/39), 17.72% (45/254), 14.81% (8/54), 11.48% (7/61), and 11.30% (20/177) in Indonesia, India, Italy, China, Germany, and Spain, respectively.^98^

In the same year, Halstead and coworkers in the UK studied the molecular ecology and transmission dynamics of *P. aeruginosa* both clinical and environmental isolates (120 clinical and 432 water) in four capital national health service hospitals. The clinical samples were from 78 patients including respiratory samples (50%), swabs (25.4%), urine (11.2%), blood cultures (6.7%), tissues (4.2%), and fluids (2.5%). They cultured pre-flushed samples on nalidixic acid agar plates supplemented with either cysteine lactose electrolyte-deficient or cetyl trimethyl ammonium bromide, and used MALDI-TOF-MS to detect the oxidase-positive colonies. Integrating both the WGS and bioinformatics data concluded non-outbreak transmission of *P. aeruginosa* from water outlets to patients, particularly in the newest hospital, which showed the highest outlet infection rates.^99^

Li and colleagues reviewed in 2024 reports on VAP-causing MDR *P. aeruginosa* in the period of 2012-2022 from 16 countries including the USA, Belgium, Brazil, China, Croatia, Germany, Greece, India, Iran, Italy, Japan, Serbia, Spain, and Turkey. Although data insufficiency, this systematic review concluded Iran to have the highest prevalence (87.5%), followed by China (85.3%), whereas the USA showed the lowest (19.7%). Based on data from 11 European countries, they concluded the prevalence of VAP caused by MDR *P. aeruginosa* in Europe to be 29.9%, where Spain showed the highest prevalence (65.9%), and Croatia showed the lowest (22.2%). Of note, such Spanish record exceeded the one which came out between 2014 and 2017, which conflicts with the European Centre for Disease Prevention and Control’s 2022 report which claims a stepwise decrease in the prevalence of MDR *P. aeruginosa* from 13.4% to 12.1% in the EU countries between 2016 and 2020.^100^

The Canadian Ward Surveillance (CANWARD) study is a national surveillance program aiming to track both bacteria inducing HAIs and their AMR longitudinally over multi-year studies.^101^ In 2025, the CANWARD study reported (371/1725; 21.5%) CR *P. aeruginosa* isolates between 2018 and 2023. The majority of these resistant strains were respiratory isolates from males aged between 18-65 years. WGS results showed broad genetic diversity with 98.1% of the isolates carried at least one of the class D β-lactamase genes such as OXA-2, OXA-5, OXA-10, or OXA-50-like subfamily, whereas the carbapenemase gene was seen only in  three isolates.^94^

##### 4.2.4.2 Regionally

In the Mediterranean region, the first recorded pneumonia case carrying the *bla*_*VIM*_ MBL gene was in Italy in 1997.^102^, ^103^ The β-lactamase assay was done spectrophotometrically and its molecular characterization was done by DNA sequencing.^103^ In Tunisia, ESBL *P. aeruginosa* carrying the SHV gene was reported in 1999^104^; and *P. aeruginosa* isolates carrying the *VIM-2* gene were detected in 2002, which were expanded thereafter as later.^88^, ^105^ The analysis was initially done by the EDTA disk synergy technique then confirmed by conventional PCR and sequencing using previously published primers.^105^

##### 4.2.4.3 Locally

**Figure 4:**
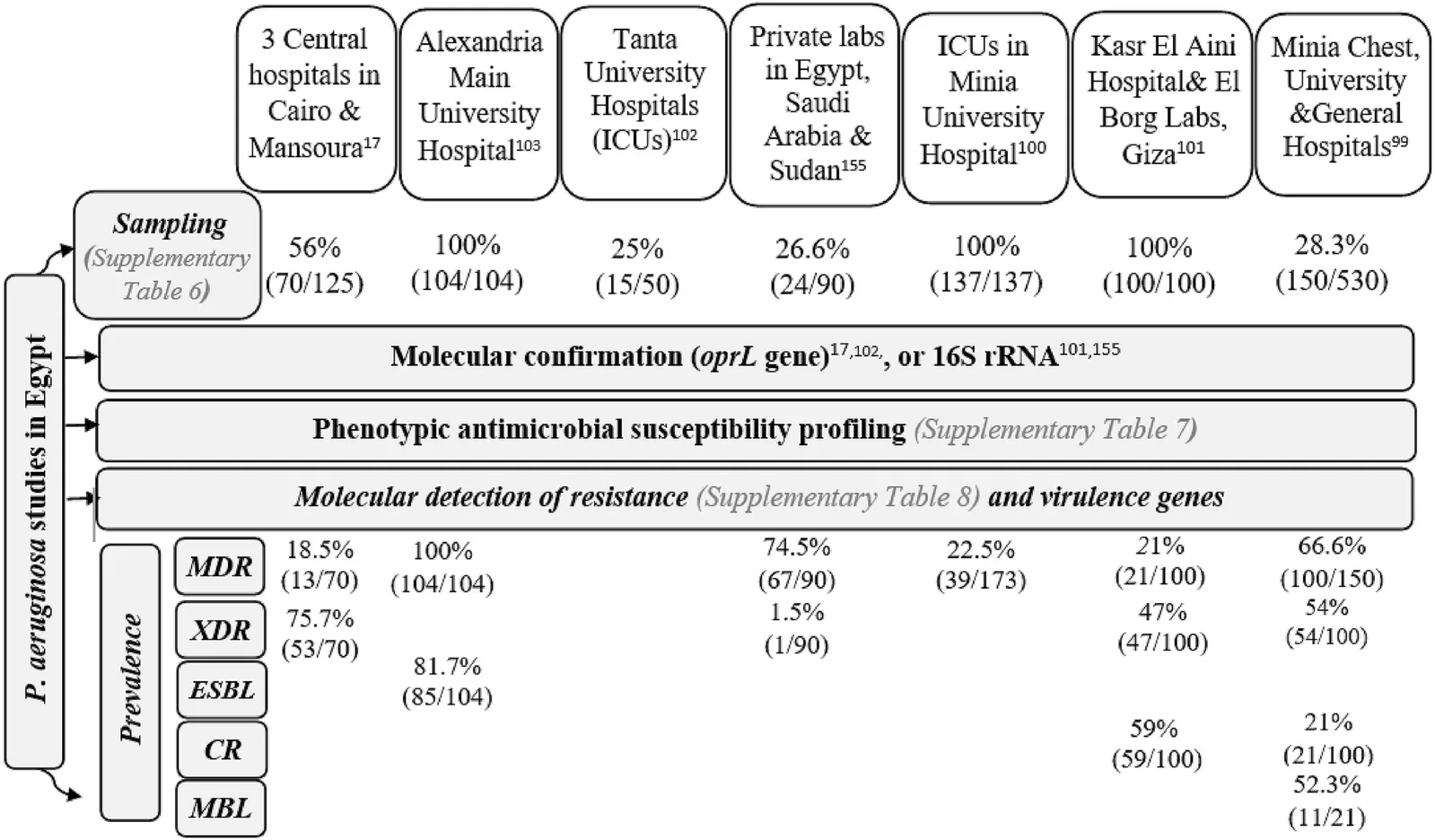
Flowchart of recent *Pseudomonas aeruginosa* investigations in Egyptian studies. *P= Pseudomonas;* MDR= Multidrug resistant; XDR= extended drug resistant; ESBL= extended spectrum β-lactam resistant; CR= Carbapenem resistant; MBL= Metallo-β- lactamase producers.

Recently, different studies were conducted in Egypt on *P. aeruginosa* in a customized narrow research scale among multiple governorates, including Cairo, Alexandria, Suez, Kafr Elshaikh, Benisweef, Fayoum, and others *(Supplementary Table 6*). These studies investigated the prevalence of *P. aeruginosa,* the emergence of MDR and XDR strains, susceptibility to different antibacterial classes, and the existence of AMR and virulence genes *(*Figure 4*).*

In Egypt, a team in 2014 identified *bla*_*NDM-1*_ with *bla*_*VIM-2*_ *P. aeruginosa* on the genotypic level from sputum and wound specimens from 2 hospitalized patients in Kasr Al Aini Hospital.^106^ The Identification was based on primers from previously published references.^107^ Isolates confirmation and antibiotic susceptibility were done by the VITEK 2 and E test, respectively.^107^

In fact, this study opened the door for further investigations of *P*. *auresignosa* in Egypt in several regions *(Supplementary Table 6)* depending primarily on phenotypic detection methods based on conventional biological methods such as culture on MacConkey and cetrimide agar plates, oxidase, sulfide–indole–motility urease, and triple sugar iron tests,^14^, ^23^, ^81^, ^108^ followed by the molecular confirmation of *P. aeruginosa* either by PCR analysis of *oprL* using previously published primers or by the 16S RNA sequencing.^23^, ^108^, ^109^ Marey and colleagues demonstrated 100% specificity, sensitivity, and reliability of the multiple cross displacement amplification assay using specifically designed primers based on GeneBank nucleotide sequences, which were manufactured in South Korea for the *P. aeruginosa oprL* gene as a rapid alternative to the routine phenotypic methods.^109^

Furthermore, the phenotypic profiling of the antibiotic susceptibility using the traditional disk diffusion method reported resistance against different classes, such as penicillins, aminoglycosides, carbapenems, and cephalosporins, across *P. aeruginosa* isolates from different regions in Egypt *(Supplementary Table 7)*. Worth mentioning, colistin recorded the minimal resistance compared to other antibiotics in two independent studies in Minia and Alexandria, respectively.^14^, ^110^ These findings contradict those of others who reported 92.86% (out of 70 isolates) as colistin-resistant *P. aeruginosa* strains in both Cairo and Mansoura *(Supplementary Table 7).*^23^

The antibacterial resistance of the Egyptian strains was confirmed by further molecular characterization of various ESBL-encoded genes, including the class A (cefotaximase *CTX-M15, bla*_*PER*_*, bla*_*PSE*_*,* and *bla*_*VEB-1*_), class B (*bla*_*IMP*_*, bla*_*NDM*_*, bla*_*AIM*_*, bla*_*VIM*_*, bla*_*GIM*_*,* and *bla*_*SPM*_), and class D (*bla*_*OXA-10*_*)* ones.^14^, ^110^

In 2019, Farhan and colleagues recorded the 28.3% (150/530) prevalence of *P. aeruginosa* among bacterial isolates from three central hospitals in Minia in which 66.6% (100/150) were MDR strains. Meanwhile, the ESBL producers and CR strains recorded 54% (54/100) and 21% (21/100), respectively (*Supplementary Table 6)*. Among the ESBL-resistant strains, they observed 55.5% (30/54) prevalence of *CTX-M15*. Additionally, other resistance encoded genes were investigated in the same study, including *bla*_*IMP*_*, bla*_*VIM*_*, bla*_*GIM*_*, and bla*_*SPM*_ by 42.8% (n=9), 52.3% (n=11), and 52.3% (n=11), 38% (n= 8) out of the twenty-one CR isolates (*Supplementary Table 8*).^14^

Furthermore, another Egyptian study conducted in 2020 by Basha and coworkers reported the emergence of CR *P. aeruginosa* strains with a prevalence 59% (59/100) of clinical isolates from Kasr El Aini Hospital and El Borg Laboratories in Giza, using Kirby-Bauer disk diffusion technique. The XDR and MDR strains were distributed in 47%, 21% of the total 100 *P. aeruginosa* isolates*;* specifically, by 79.66% and 20.33% across 59 CR strains, respectively *(Supplementary Table 6*). Additionally, they observed the individual existence of *bla*_*NDM-1*_ and *bla*_*VIM-1*_ resistance genes in 11 isolates by 90.9% (n=10) and 18.1% (n=2), while the gel electrophoresis presented their co-existence in only one isolate. Notably, they reported complete absence of both *bla*_*IMP*_ and *bla*_*VIM-*2_ alleles among their isolates *(Supplementary Table 8).*^108^

In 2024, Abdelaziz and coworkers reported the existence of XDR and MDR *P. aeruginosa* strains in isolates from various hospitals in Cairo and Mansoura with prevalence of 75.70% (53/70) *and* 18.50% (13/70), respectively *(Supplementary Table 6)* where they highlighted *the oxa* gene as the most frequently CR gene among these isolates by 91.4% (64/70), while other ESBL resistance genes such as *VIM-1, NDM-1* and *VIM*-*2* were recorded in 74.3% (n=52), 45.7% (n=32), and 21.4% (n=15) across the 70 clinical isolates, respectively. In addition to the ESBL and CR-encoded genes, Abdalaziz’ team expanded their investigation to record other resistance genes that encode aminoglycoside resistance including *aac(6′)-lb, aac(3)-II, rmtB, aph (3’)-I* by 84.30% (59/70), 81.40% (57/70), 74.30% (52/70), and 31.40% (22/70), respectively. In their hands, the *mcr-1* gene which encodes the colistin resistance recorded the lowest percentage (12.9%; 9/70) among their *P. aeruginosa* isolates (*Supplementary Table 8)*. Notably, these molecular analyses were conducted by conventional PCR analysis using primers from previously published references.^23^

Alongside, Edward and colleagues had reported the emergence of ESBL encoding genes in 81.7% (n=85) out of 104 clinical *P. aeruginosa* isolates from Alexandria Main University Hospital (*Supplementary Table 6)*, of which 69 isolates harbored a single resistance gene. Upon plasmid profiling, they highlighted the co-harboring of more than one ESBL-encoded gene, including double combination of *bla*_*OXA-10*_ with *bla*_*PER*_*, bla*_*VEB-1*_, or *bla*_*PSE*_ within 7.7% (8/104), 5.8% (6/104), and 1% (1/104), respectively; or in triple co-existence of *bla*_*OXA-10*_ with both *bla*_PER_ and *bla*_VEB-1_in only one isolate. They also reported the co-existence of ESBL and MBL in a double combination of *bla*_*OXA-10*_ with *bla*_*VIM*_ *or bla*_*AIM*_ by 1% (1/104) and 1.9% (2/104), or in triple combination with *bla*_PER_ and *bla*_*AIM*_ in only one *P. aeruginosa* isolate. Notably, they observed the MBL-encoded gene in all imipenem-resistant *P. aeruginosa* strains.^110^

On the other hand, others reported the existence of some virulence genes in MDR *P. aeruginosa* isolated from different ICUs in Minia University Hospital including GDP-mannose dehydrogenase enzyme for alginate (*algD*), exoenzyme S and U (*exoS* and *exoU*), exotoxin A (*toxA*), alkaline protease (*aprA*), elastase (*lasB*), and hemolytic and non-hemolytic phospholipase C (*plcH* and *plcN*) by 30.7% (n= 12), 51.3% (n= 20), 50.4% (n= 22), 23% (n= 9), 67.2% (n= 27), 43.5% (n= 17), 12.8% (n= 5), and 0% (n= 0) of 39 MDR isolates, respectfully.^81^

### 4.3 Staphylococcus aureus (S. aureus)

#### 4.3.1 Classification and pathogenesis

It is Gram-positive anaerobic bacteria naturally present as a normal flora of the skin, the upper respiratory tract, the intestine, and the vagina,^111^, ^112^ where any changes in normal conditions like temperature, pH, or media composition can affect its nature and make it pathogenic.^111^ It can cause localized infections such as cutaneous folliculitis or more serious pathogenesis, including pneumonia, food poisoning, endocarditis, and bacteremia.^111^, ^113^ Thus, *S. aureus* is regarded as a health-threatening CAI-, HAI-, and livestock-associated infections-causing pathogen.^108^, ^112^, ^114^ The systematic review and meta-analysis study of the Antimicrobial Resistance Collaborators reported that *S. aureus* was deadly among 135 countries, where it was implicated in more than 1 million deaths and 34 million years of life loss, globally. Yet there is no global public health investment directed to *S. aureus.*^8^ The majority of research data about *S. aureus* in the Middle East, Africa, and Egypt are either veterinary or zoonotic, i.e. livestock-human contact-related.^108^

The genus *Staphylococcus* was first discovered by the surgeon Alexander Ogston in 1870, who observed post-surgical death of many patients.^15^, ^115^ Upon staining pus isolates by the aniline violet dye, *Staphylococcus* was seen under light microscopes as a round organism.^15^, ^115^ Ever since, *S. aureus* has been considered as one of the major causes of both HAI and CAI in various countries including Canada, Denmark, the Netherlands, Finland, and Norway, which causes pneumonia, bacteremia, endocarditis, sepsis, toxic shock syndrome, and other life-threatening conditions.^116^, ^117^, ^118^, ^119^

Among community-acquired methicillin-resistant *S. aureus* (CA-MRSA), the ST8 (USA300) and ST1 (USA400) lineages have been the most predominant over different regions, including the USA and Canada. Across other regions, CA-MRSA is mostly caused by ST8 in Europe, ST72 in Korea, ST59 in Vietnam, Taiwan, and China.^120^, ^121^, ^122^, ^123^

The Staphylococcal cassette chromosome mec (SCCmec) is a transferable element (called a genomic island) that carries the *mecA* gene.^32^, ^124^ There are more than ten types of SCCmec; however, according to the International Working Group on the SCC, the main types of SCCmec are distributed into IV/V/VII, which are related to CA-MRSA, while I/II/III are mostly associated with hospital-acquired MRSA.^32^, ^114^, ^124^, ^125^ Type IV SCCmec is the most common among CA-MRSA isolates and the smallest in size, which makes it the most transferable form.^114^

Furthermore, the panton-valentine leucocidin (PVL) and toxic shock syndrome-toxin 1 are virulence genes associated with MRSA.^32^, ^126^ Unfortunately, PVL is mostly associated with CA-MRSA; meanwhile, positive MRSA could lead to severe complications such as wrecking of leukocytes and necrosis of soft tissues in the case of necrotizing pneumonia or skin infections.^32^

#### 4.3.2 Diagnosis

By Gram staining alongside its morphological characterization, growth on selective media such as blood or mannitol salt agar, as well as other biological tests, including coagulase, modified oxidase, and DNase tests.^112^, ^113^ Further confirmation of *S. aureus* is done by PCR amplification of the *nuc* gene.^113^ In addition to the *nuc* gene, the phenotypic characterization of MRSA strains was confirmed by PCR amplification of the *mecA* gene.^119^, ^127^ The USA400 was the first recorded deadly MRSA strain for many pediatric patients in the USA.^128^, ^129^

#### 4.3.3 Therapy

Treatment options were limited to topical phenolic acid or other antiseptics until penicillin was discovered in the 1940s.^130^, ^131^, ^132^ Disappointingly, the first observation of penicillinase-producing *S. aureus* was within two years of penicillin usage.^120^, ^132^ By 1950, resistance to penicillin became more frequent; which encouraged developing new antibiotics.^132^ Consequently, methicillin was discovered as a semi-synthetic alternative β-lactam antibiotic, but as expected, MRSA strains were developed by 1961.^132^, ^133^ The MRSA expanded causing endemic outbreaks in various regions, e.g. the USA outbreak by the end of the 1960s.^129^, ^134^, ^135^ and 50 000 annual deaths both in Europe and USA.^136^ The identification of MRSA was done by both conventional and standard techniques for antimicrobial susceptibility according to the Clinical and Laboratory Standards Institute (CLSI) guidelines.^119^

#### 4.3.4 Prevalence

##### 4.3.4.1 Globally

In 2019, a systematic review and meta-analysis study that included 38 studies of 864 *S. aureus* isolates from 22 different countries (including India, China, Pakistan, and other countries) over 10 years was conducted, where *S. aureus* strains were found to be equally distributed between tropical (64%, 112/173) and non-tropical (67%, 466/691) regions. Additionally, 67% (577/864) of these isolates were methicillin resistant, distributed among high-, upper-, and low-middle-income countries in 69% (220/319), 67% (331/493), and 50% (26/50), respectively.^41^

In 2023, Adhikari and coworkers reported 6.71% (499/7433) positive *S. aureus* among clinical specimens from a tertiary teaching hospital in Nepal. They identified *S. aureus* based on colony’s morphology, Gram staining, catalase-, and coagulase-test. Also, they applied the Kirby-Bauerdisk diffusion method for estimation of strains’ susceptibility to antibiotics and recorded 26.4% MRSA isolates, which were mostly MDR (94.05% of MRSA) present mainly in pus (71; 18.5%). The rest MRSA were distributed among blood, sputum, body fluid, and urine by 12 (3.1%), 8 (2.1%), 7 (1.8%), and 3 (0.8%), respectively. All MRSA isolates were absolutely resistant to both penicillin and cloxacillin, less resistant to ciprofloxacin, erythromycin, cephalexin, cotrimoxazole, and clindamycin (80.5%, 79.8%, 64.9%, 61.1% and 58.5%, respectively) and the minimal resistance was seen in the case of both gentamicin and chloramphenicol (27.4%, 17.9%, respectively), yet, all were 100% sensitive to vancomycin.^137^

In 2024, other team collected 300 throat and nasal swabs alongside hygiene questionnaires from emergency medical services workers in both Cologne and Greifswald, Germany between 2019 and 2021 due to their frequent contact with carrier patients of both methicillin-susceptible *S. aureus* (MSSA) and MRSA strains which increases their probability to acquire the infection and transmit it to their surroundings, including patients. Although the PCR results reported a low prevalence of MRSA (1%), which aligned with that of the total German population, the MSSA was higher than expected (43.7%). However, poor hand hygiene was significantly related to the colonization of the MSSA, particularly in males.^138^

##### 4.3.4.2 Regionally

In 2023, Kmiha and colleagues isolated 64 MRSA from blood, sputum, catheters, nasal pus, sebum, and catheters from the Traumatology and Burns Center at Ben Arous in Tunisia, in which they had initially identified *S. aureus* by both conventional and biochemical methods then confirmed the results by PCR amplification of the *nuc* gene using previously published primers as well as the *S. aureus* (ATCC 43300) strain as a control. Using previously published primers in a Canadian study, PCR results revealed presence of the *mecA* gene in approximately 78% (50/64) of all MRSA isolates.^139^

##### 4.3.4.3 Locally

**Figure 5:**
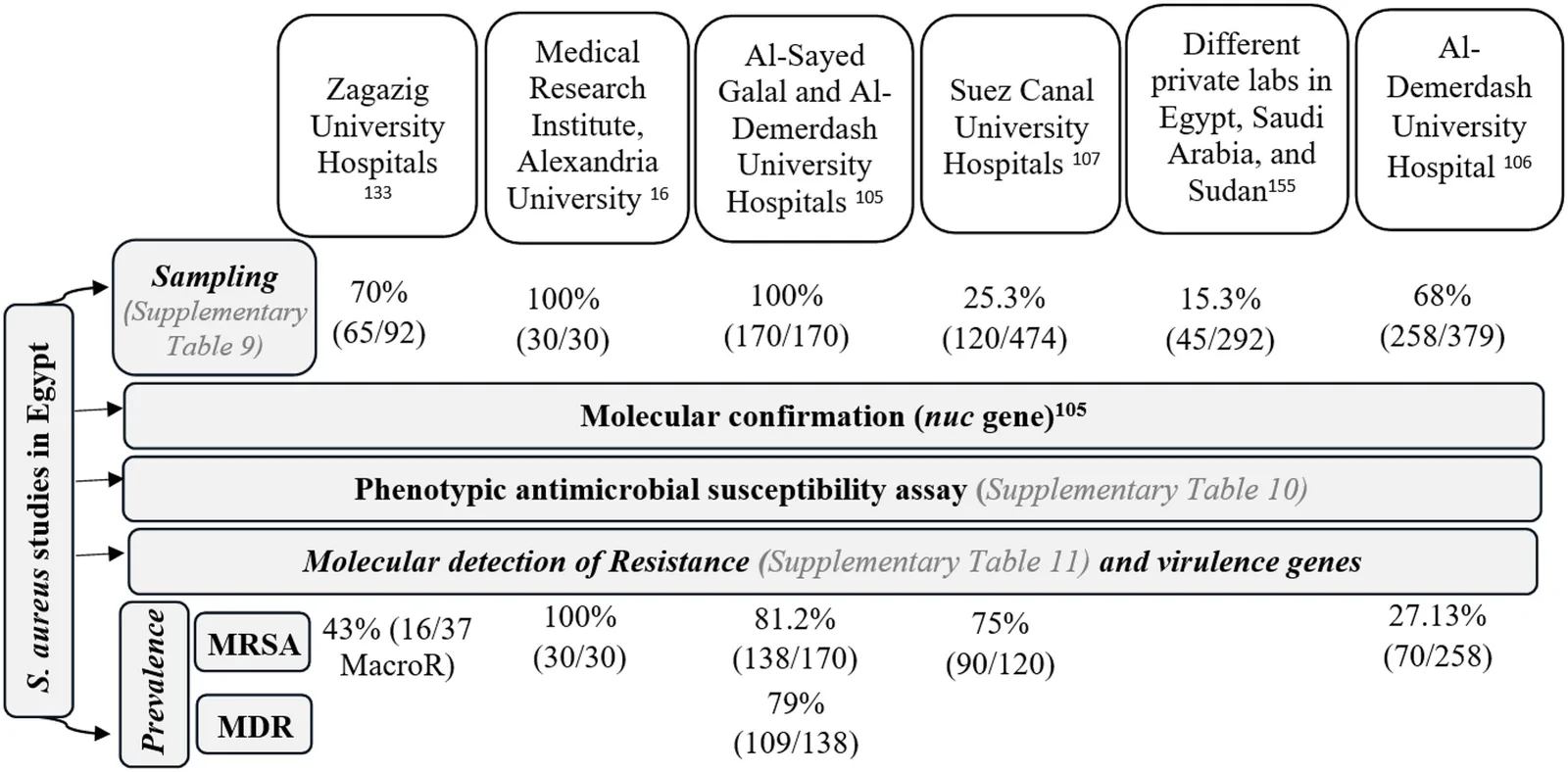
Flowchart of *Staphylococcus aureus* investigations in Egyptian studies. *S. aureus= Staphylococcus aureus;* MRSA= methicillin-resistant *S. aureus*; MacroR= macrolide resistant strains; MDR= multi-drug resistant.

In Egypt, limited studies were conducted to study S. aureus and their MRSA strains (Figure 5, Supplementary Table 8); the majority relied on the above mentioned conventional methods for S. aureus identification.^112^, ^113^

The prevalence of *S. aureus* in both Al-Sayed Galal and Al-Demerdash University Hospitals in wound swabs, blood, intravenous catheters, sputum, urine, pus, abscesses, eye swab, intra-tracheal aspirate, and urinary catheters were 34.1% (58/170), 15.3% (26/170), 10% (17/170), 10% (17/170), 8.8% (15/170), 8.8% (15/170), 5.9% (10/170), 5.9% (10/170), and 1.2% (2/170), respectively *(Supplementary Table 9).*^113^

In 2020, the prevalence of *S. aureus* in Al-Demerdash Hospital among various Gram-positive isolates (n= 379) was reported to be 68% (n= 258), where it mainly existed in urine, pus, throat swabs, and sputum with percentages 33.3, 40, 12.8, and 1.2, respectively. Of the 258 *S. aureus* isolates, 70 were MRSA in upon using the oxacillin resistance screening agar base medium plate.^112^ Using the cefoxitin by the disk diffusion technique, another team in the Zagazig University Hospitals detecting 61.5% (n= 16) MRSA among 26 macrolide-resistant *S. aureus* isolates from the ICU *(Supplementary Table 9*).^140^

Four studies were conducted across several Egyptian hospitals to detect the antimicrobial susceptibility of *S. aureus* isolates using the conventional disk diffusion method following guidelines of the CLSI (*Supplementary Table 10*) where amikacin (one of the aminoglycosides that has observed high resistance rates) was recorded resistance by 77.1% (54/70), 53.3% (16/30*)*, and 31.8% (21/66 *PVL*-) among the MRSA isolates in Al-Demerdash-, Research Institute in Alexandria-, and Suez- University Hospitals’ studies, respectively.^22^, ^112^, ^114^ In other study, the prevalence of MRSA in both Al-Sayed Galal-with Al-Demerdash -University Hospitals was 26.1% (36/138).^113^ In addition, high resistance rates were seen for the clindamycin and erythromycin (members of lincosamide and macrolide families, respectively), especially in the study of Ibrahim’s team***.*** In contrast, the highest sensitivities were recorded upon testing both linezolid and rifampicin *(Supplementary Table 10).*^22^, ^113^, ^114^

These Egyptian studies often confirmed the *S. aureus* diagnosis by PCR amplification of the *nuc* gene. Alfeky and colleagues confirmed 100% (n= 170) of *S. aureus* from both Al-Sayed Galal and Al-Demerdash University Hospitals using primers based on Iranian references.^113^ In addition to the *nuc*, the *mecA* gene was also used for the molecular confirmation of the MRSA strains by PCR. PCR for the *mecA* gene based on previously published primers in Canadian references among *S. aureus* isolates from Al-Sayed Galal & Al-Demerdash University Hospitals, and the Suez Canal University Hospitals revealed MRSA prevalence rates of 81.2% (138/170) and 75% (90/120), respectively *(Supplementary Table 11).*^113^, ^114^ Others investigated the erythromycin ribosomal methylase (including *ermA, B,* and *C*) and macrolide-streptogramin resistance (*msrA*) genes by PCR using published primers in a French study among isolates from Zagazig University Hospitals and reported 16.2% (6/37), 5.4% (2/37), 54.1% (20/37), and 45.9% (17/37) detection rates, respectively.^140^, ^141^, ^142^

Moreover, Egyptian studies included detection of virulence genes such as the PVL gene in MRSA isolates by PCR.^114^ In El-Sweify and coworkers’ study, the PVL gene was detected in 26.7% (24/90) of MRSA isolates from Suez University Hospital using published primers by the Duke University Medicinal Center in North Carolina and the *S. aureus* NCTC13300 strain as a control. Additionally, they used the QIAGEN® Master Mix of four primer sets to investigate SCCmec gene types II, IV, and V, which were distributed among PVL-positive strains by 8.3% (2/24), 75% (18/24), and 8.3% (2/24), respectively; while the remaining 8.3% were non-typeable.^114^ On the other hand, Monecke *et al.* recorded 40% (12/30) PVL*-*positive *S. aureus* across their isolates from Alexandria.^22^

### 4.4 Factors affecting the quality of surveillance data

The burden of bacterial pneumonia is still undefined in many localities in the world, specifically in LMICs, which hinders the assessment of pneumonia’s incidence and prevalence.^10^, ^13^, ^42^ Even available global tracking systems for AMR bacterial infections are confronted with short, under-sampled, and fragmented data for estimation.^13^ However, the growth of AMR among respiratory bacterial strains has reviewed to be increased recently, especially after the COVID-19 pandemic^143^ due to the following factors:

### 4.5 Population awareness

Fadl’s team reported that the population's socio-cultural and awareness status play a critical role in bacterial infection and resistance development. Mothers’ education and awareness assist pneumonia management in children under 5 years. Low educational level in resource-limited countries makes them unaware of the causes, symptoms, and complications of pneumonia. Thus, they ignore seeking medical consultation and the proper child-healthcare practices. Additionally, the limited experience of handling antibiotics at home may exacerbate complications.^144^

Additionally, in rural communities, self-medication and misrepresentation of antibiotics are common owing to their availability and easy accessibility.^136^, ^143^, ^145^ Furthermore, 17% of patients often left-over antibiotics upon initial relief of symptoms, and the remaining pills are commonly recommended to other family members without completing the prescribed therapeutic regimen.^136^

### 4.6 Level of Healthcare facilities and personnel

Many healthcare providers in community pharmacies are unskilled or undertrained, particularly in LMICs of Africa, Asia, and Latin America, such as India and Egypt.^136^ The insufficient awareness allows them to dispence antibiotics for common cold, sore throat, or cough without physicians’ prescriptions.^136^, ^145^ These professional misconducts drive the aggravation of bacterial resistance against antibiotics. Consequently, it leads to high therapeutic burden for both individuals and governments due to worsened health-related complications and prolonged hospitalization,.^40^, ^146^, ^147^

Disappointingly, it was reported that more than 50% of hospitals cannot afford the Gram staining for bacterial specimens in LMICs.^8^ Limited hospitals can apply cultures, antimicrobial susceptibility, as well as genomic assays.^8^, ^13^, ^136^ Additionally, conventional diagnostic strategies require well-trained personnel and consume at least 48 to 72 hours, which is considered time-waisting.^12^ As a result, many physicians are forced to diagnose based on clinical manifestations, regardless of the confirmatory laboratory measures.^136^ In turn, they initiate an empirical therapy with antibiotic prescriptions, leading to expansion of AMR strains and hinders the desired therapeutic outcomes.^40^, ^136^, ^148^

Furthermore, deviations from therapeutic guidelines and infection control frameworks in healthcare facilities due to prophylactic or therapeutic overuse of antibiotics, poor sanitation, and inadequate hygiene of healthcare workers, close patient-to-patient interactions, results in the exacerbation of AMR and HAI.^136^

### 4.7 Level of administration and governance

Surveillance programs in developing countries are not successively tracking the prevalence of bacterial infections and their resistance patterns among various WHO regions, including Africa, Southeast Asia, and the Eastern Mediterranean region.^11^ Closed systems, shortage of trained healthcare manpower, poor laboratory infrastructure, and outdated technologies hinder the updating of domestic surveillance profiling of VAP, HAI and AMR.^13^, ^136^ Additionally, the limited resources contribute in hindering the implementation and adherence to both national and global frameworks for AMR and infection control policies.^13^, ^136^ Altogether are leading to an epidemiological gap for heavy-burden bacterial-resistant countries.^136^ On the other hand, findings ensured that the openness of the communities and political turbulence assisted cross-regional expansion of MDR bacterial strains.^149^ Thereby, the demographic, geographic, and socio-economic levels are terrorized irrespective of national income levels.^11^

## 5 Regulatory aspects

Numerous regulations are issued to control infection and antimicrobial prescription either in hospitals or pharmacies. Hospital-wide, infection control and accreditation strategies are obligatorily implemented all over the world. These policies are targeting the prevention of nosocomial infections either by frequent handwashing, alcohol-based hand rub outside patient rooms, or glove renewal upon interaction with individual patients.^136^ These interventions limited the development of cross-contamination inside the medical facilities, while the progress of AMR can be controlled through appropriate stewardship strategies for antibiotic use which leads to less antibiotic consumption, shorter duration of treatment, and fewer complications and side effects.^136^

## 6 Organizations’ and committees’ efforts

The WHO pointed out bacteria of concern to be targeted for monitoring and development of new effective therapies through both: the global antimicrobial resistance and surveillance system (GLASS), and the bacterial priority list *(Supplementary Table 1).*^19^, ^65^ *ESKAPEE* pathogens refer to *Enterococcus faecium, S. aureus, K. pneumoniae, Acinetobacter baumannii (A. baumannii), P. aeruginosa, Enterobacter spp, and Escherichia coli* that are major drivers of HAIs and demonstrate a significant proportion of drug resistance globally.^150^ Recently, the Gates and Novo Nordisk foundations adopted the Gram-negative antibiotic innovation project targeting combating AMR strains that are associated with health risks, especially in LMIC. This program supports research for novel antibacterial agents, alternative therapies, and screening tools to address AMR, especially for the Gram-negative *Enterobacteriaceae* family including *K. pneumoniae.*^151^

Numerous national action plans have been enacted among the WHO-Eastern Mediterranean zone and the Arab Gulf states in answer to the Global Action Plan (GAP) for AMR control, supporting the one health strategy. Gulf Countries including Bahrain, Qatar, Kuwait, Saudi Arabia, the United Arab Emirates, and Oman incorporated multidisciplinary efforts between joint sectors, either through governmental authorities or private entities, as core components of their action plans^34^ the Egyptian action plan (2018-2022) has been issued for controlling AMR by the Egyptian Ministry of Health and Population.^152^ It included four main purposes: 1) improving societal awareness, 2) optimizing the consumbtion of antibiotics to manage the expansion of resistant bacteria and mitigate the emergence of new resistance patterns, 3) improving the domestic surveillance program to combat resistant strains under the umbrella of the integrated Health approach, and 4) operationalizing an evidence-based infection control measures. Moreover, it highlighted the weakness points that hamper national goals, involving poor infrastructure of microbial labs, limitation of experimental setup, lack of antibiotic use surveillance, shortage in data sharing, and cross- and within-sectorial communication in both human and vetereinary domains.^152^

Also, the Committee of National Rational Antimicrobial Use in the Egyptian Drug Authority (EDA) has pursued actions to control antibiotic resistance. They issued in 2024 the national guideline for antimicrobial use in infections with MDR pathogens.^153^ In this guideline, they concerned with the ESKAPE pathogens as the most critical AMR organisms causing serious infections in medical facilities such as pneumonia and bacteremia. Additionally, they recommended empirical antibiotic combinations for each AMR strain in various infections. Furthermore, in the same year, the EDA published the antibiotic prescription framework for preventing over-the-counter dispensing of reserve antibiotics,^154^ which are listed by Health Canada and WHO as last-choice in confirmed or suspected MDR bacterial infections only.^155^

## 7 Discussion

Herein we emphasized *K. pneumoniae*, *P. aeruginosa*, and *S. aureus* as repeatedly engaged with HAI or CAP. The prevalence of these infections varies from across sites based on finantial, cultural, regulatory, and governance factors that lead to AMR developing, mainly in LMICs.

In fact, developed countries are impacted by other factors, such as the widespread prophylactic use of antibiotics in chemotherapy or common surgery.^136^ Moreover, traveling to districts where AMR species are epidemic or endemic, and the antibiotic implimentation in food-producing animals might accelerate the speed of drug-resistant strain propagation. In turn, AMR is a significant public health menace that threatens communities all over the world, neglecting the income level.^136^

In parallel, we highlighted the global and regional shortage in epidemiological and surveillance findings of pneumonia. Likewise, Egypt has conducted a range of pilot-scale research across various sites. Many factors limited these studies, including small sample sizes, diverse socioeconomic and cultural backgrounds, inequality of medical facilities and laboratory capabilities, as well as the absence of standardized sampling and diagnostic methodologies.

Nevertheless, nearly all these studies presented the existence of these three pathogens with moderate to high prevalence, lay between 10% and >50% of the total isolates. Resistance against carbapenem and other β-lactam antibiotics is extensively showed among *K. pneumoniae* and *P. aeruginosa* strains. These records agreed with Azab’s team*,* who compared prevalence rates in Egypt, Sudan, and Saudi Arabia which were 17.8, 17.5, and 14.8% for *K. pneumoniae*, and 26.7, 42.5, and 25.9% for *P. aeruginosa.* In the same study, the burden of *S. aureus* were 18.9 and 17.3% among Egyptian and Saudian isolates, respectively.^156^ The study reported the prevalence of *K. pneumoniae, A. baumannii, and P. aeruginosa* in Egypt from 2014 to 2015 among tracheal aspirates by 36.9, 14.95, and 14.16%, respectively.^157^

In Egypt, outcomes of the time-consuming conventional bacterial differentiation and antibiotic susceptibility tests indicated high resistance to ampicillin, penicillin, ampicillin/sulbactam, and amoxicillin/clavulanic (*Supplementary Tables 3, 7, and 10)*. Additionally, pneumonia-causing bacterial specimens showed elevated resistance rates to carbapenems and quinolones, while the least resistance rates were observed with colistin (*Supplementary Tables 3, 7, and 10)*. These results are aligned with the review of El-Kholy’s team*,* which revealed the high rates of ESBL resistance among *K. pneumoniae*, ranged 10 - 87%. Moreover, they noted the frequency of CR *K. pneumoniae*, *P. aeruginosa*, and *A. baumannii*, ranges of 35 - 100%, 15 - 70%, and 10 - 100%, in Egyptian hospitals between 2004 and 2020.^45^

Considering molecular analysis, it allows fast specific diagnosis of pathogens and controls the empirical treatment of broad-spectrum antibiotics for bacterial pneumonia.^157^ However, our findings highlighted the presence of many carbapenem and ESBL antibiotic resistance genes in isolates from different localities in Egypt, including *VIM, OXA-48*, *NDM,* and *CTX-M-15.* Also, the *mecA* gene is mostly implimented in the detection of methicillin resistance in *S. aureus*. Several studies also included genotypic recognition of virulence genes such as PVL in *S. aureus* and exoS, algD, toxA, and exoU *in P. aeruginosa*. Furthermore, the co-harboring of double or more resistance genes was reported across strains, such as *bla*_*NDM*_ with *bla*_*OXA-48*_ in *K. pneumonia*, *bla*_*VIM-1*_ with *bla*_*NDM-1,*_^108^ *bla*_*OXA-10*_ with *bla*_*PER*_*, bla*_*VEB-1*_*,*or *bla*_*PSE*_ among *P. aeruginosa*^72**,**101**,**103^. Notably, PCR is not enough to confirm the co-localized of resistance genes, however, we need further confirmation by other molecular profiling, such as WGS, plasmid profiling, and expression or enzyme-activity. This discrepancy between PCR-only, WGS, and plasmid-confirmed gene co-existance reflects a broader challenge across many healthcare facilities, particularly in LMICs. Limited laboratory resources often preclude further confirmatory molecular techniques. Therefore, the reliance on partially- or un-confirmed diagnostic data contributes to gaps in local surveillance accuracy, which in turn hinders the implementation of precise, evidence-based antimicrobial stewardship guidelines and appropriate empirical therapy prescription.

Regrettably, Assef reviewed that the interpretation of molecular diagnostic techniques might differ from the local treatment recommendations and actually existing resistance genes, causing false-negative outcomes. Therefore, DNA sequencing can further validate the PCR detection of pathogens.^157^ Sequencing patterns can assess the identification of host response and broaden the therapeutic potential for research targeting personalized therapeutic approaches and stewardship prescription of antibiotics, which has not yet been observed, specifically in local studies, particularly in LMICs. These same limitations directly constrain the development of local treatment guidelines informed by locally adapted antibiograms, leaving well-trained clinicians reliant on international recommendations, including WHO-based guidance, that may not accurately reflect the local epidemiology of AMR.

Since the WHO affirmed that AMR is a worldwide health issue in 2000, countries across the global began to align with international frameworks to initiate strategies seeking to controlling this threat.^152^ Further, the WHO implemented the GAP and the GLASS in 2015.^152^, ^158^ However, even the latest amendments of British Thoracic Society “BTS 2011” and Infectious Diseases Society of America “IDSA 2019” protocols for pneumonia management did not recommend microbial investigations as a routine measurement for CAP except in severe and complicated conditions because of the low quality of conventional laboratory methods.^159^, ^160^ Alongside highlighting the absence of precise diagnosis in these guidelines, the first-line empirical therapy for severe cases, such as levofloxacin, amoxicillin, co-amoxiclav, azithromycin, cefaclor, erythromycin, clarithromycin, and macrolide,^159^, ^160^, ^161^ displayed moderate to high resistance rates in emerging Egyptian studies.

Despite the efforts of several initiatives to track the bacterial infection and resistant strains to control their expansion on either a national or global scopes, many gaps between the actual burden of bacterial pneumonia, antibiotic resistance, on-time and precise diagnosis, and precise therapeutic control still exist. These gaps refer to the insufficient socioeconomic standards, unawareness of the population, inadequate lab capacities, substandard laboratory infrastructures, limited diagnostic tools, and obsolete techniques. Furthermore, the absence of firm adherence to domestic regulations that were issued to control self-directed therapy and injudicious antibiotic prescriptions, mainly in LMICs, magnifies the antibiotic resistance progress. In addition, these limitations hinder the progress towards the development of novel and effective antimicrobial agents.

## 8 Conclusions

Bacterial pneumonia is a pivotal public health threat globally. Additionally, the AMR is a worldwide concern within the ambit of the one health approach. The growth of morbidity and fatality measures of CAP and HAP is influenced by different elements, including population enlightenment, socioeconomic status, adherence to both global and local regulations for infection control, and others. Thus far, the world has possessed a marked gap in monitoring epidemiological dynamics of bacterial pneumonia and their AMR strains, which might be attributed to inadequate advancement of precise diagnostic tools or insufficient coordination between within and across-sectors in healthcare systems.

Timely detection of bacterial pneumonia is challenged by overlapped clinical presentations with viral infection, time-consuming traditional diagnostic assays, shortage of both qualified manpower and funding for molecular profiling techniques. Even the to date molecular tools is not conclusive caused by continuous mutation of resistance genes. However, the mitigation of AMR demands a rational antibiotics administration, improving diagnosis, continuous epidemiological surveillance analysis of status, and investment in novel potential medications.

## 9 Our perspectives

Complying with infection control frameworks and antibiotic stewardship protocols, the precise and timely diagnosis of infected cases is imperative. Therefore, it is indispensable to support healthcare facilities with proper laboratory infrastructures, upgraded equipments, and detection kits to facilitate validated laboratory diagnosis of causative pathogens. To limit further expansion of AMR, clinicians should prescribe both the appropriate dose and duration of the optimal antibiotic. The incorporation of updated molecular diagnostic approaches into routine healthcare can aid the establishment of multicenter surveillance sites, especially in LMICs. Both prompt diagnosis and documentation should significantly contribute to refining epidemiological surveillance based on accurate data updating nationally and globally. Given the persistent heterogeneity in antimicrobial susceptibility testing pipelines across healthcare and research centers in Egypt and other LMICs, we recommend that the WHO propose standardized diagnostic protocols for uniform implementation by national health authorities worldwide. Such protocols should be designed for feasible adoption within LMIC settings, accounting for local resource constraints, to progressively harmonize testing methodologies and enable more accurate, comparable estimation of AMR burden across regions.

At the national level, Egypt illustrates the obstacles that face LMICs. We are confronted with the underdevelopment of molecular diagnostic kits based on domestic sequences of resistant bacterial strains. Thus, the accessibility of domestic genetic diagnostic tools for pneumonia-causing bacteria can directly lead to more robust results, stewardship dispensing of antibiotics, commitment to infection control protocols as well as both timely and accurate notifying national surveillance system.

Indeed, the outlook for antimicrobial control resides in the integration of modern approaches that extend beyond traditional detection and therapeutic strategies. Advancements in diagnostics and the increasing availability of surveillance data will facilitate the design of future meta-analytic studies. In the interim, the comparative trends reported here reflect a qualitative synthesis of available regional evidence rather than a pooled, method-adjusted estimate of true comparative antimicrobial activity in both local and global levels, which could ultimately support more informed decision-making for improved healthcare services. To illustrate, the repurposing of existing medications provides time- and cost-effective rationales for identifying new antimicrobial applications of commercially available drugs. The In-depth analysis of host–pathogen interactions can reveal novel targets to be hit, especially in view of the frequently occurring mutations within both the antibiotic resistance and virulence genes. Optimistically, the AI-driven revolutions in computational modeling and *in-silico* drug discovery permit rapid screening and optimization of promising antimicrobial ligands, accelerating the discovery journey. Jointly, these approaches are markedly promising for addressing the global challenge of AMR through validated-targeted and resource-efficient benchmarks.

## 10 Limitations

1) We justified the highlighting of these 3 key pathogens with data availability, which limits the generalizability of our conclusions regarding other bacterial and non-bacterial causes of pneumonia, AMR profiles, surveillance priorities, and empirical therapy to the broader landscape. 2) Most AMR estimates in LMICs are based on tertiary hospital data, which, together with widespread self-medication, may overestimate resistance because of referral and selection biases. These factors should be considered to improve the representativeness of AMR surveillance.

## CRediT authorship contribution statement

**Yomna Rabie Elsherif:** Writing – original draft, Data curation. **Dina Nadeem Abd-Elshafy:** Writing – review & editing. **Mahmoud Mohamed Bahgat:** Writing – review & editing, Supervision, Conceptualization.

## Declaration of competing interest

The authors declare that they have no known competing financial interests or personal relationships that could have appeared to influence the work reported in this paper.
