# Supplementary material for: Antimicrobial resistance in three pneumonia-causing bacterial species: the global, regional, and local state of the art
Source: J Genet Eng Biotechnol. 2026 Aug 21;24(4):100802. doi: 10.1016/j.jgeb.2026.100802 (PMC13524594; doi:10.1016/j.jgeb.2026.100802)
Supplement: Supplementary file 1 [file mmc1.docx]

**Supplementary Tables**

**Table 1: Ranking of the reviewed pneumonia-causing bacteria according to the WHO Pathogens priority list**^148^

| Bacteria | Antibiotic Resistance | WHO Priority rank | |
| --- | --- | --- | --- |
|  |  | 2017 | 2024 |
| *Klebsiella pneumoniae* | Carbapenem | 5 | 1 |
|  | Third-generation cephalosporin | -- | 6 |
| *Escherichia coli* | third-generation cephalosporin | 4 | 2 |
|  | Carbapenem | 10 | 5 |
| *Acinetobacter baumannii* | Carbapenem | 1 | 3 |
| *Pseudomonas aeruginosa* | Carbapenem | 2 | 10 |
| *Enterobacter species* | Carbapenem | 9 | 12 |
|  | Third-generation cephalosporin | 6 | 15 |
| *Staphylococcus aureus* | Methicillin | 13 | 14 |
| *Streptococcus pneumoniae* | Macrolide | 20 | 21 |

**Table 2: Recent studies on Klebsiella pneumoniae isolates from Egyptian hospitals**

| **Publication** | **Collection period** | **Sample**  **size** | **Sample type** | **Hospital** | **Prevalence** |
| --- | --- | --- | --- | --- | --- |
| El-kholy *et al*. 2024^41^ | Jan. 2021:  Sep. 2021 | 94 *CRKP isolates* | Sputum, blood, urine, tracheal aspirate, wound swab, broncho-alveolar lavage, and catheter | Mabaret El Asafra Laboratories, Alexandria. | 100% of isolates (94/94) are CR *KP* distributed as: blood (51), wound swab (13), tracheal aspirate (8), sputum (8), urine (8), broncho-alveolar lavage (5), and catheter (1). |
| Ramadan *et al*. 2021^72^ | Jan. 2020:  Nov. 2021 | 176 isolates | ETAs of VAP | ICU of Zagazig Uni Hospital (a tertiary referral hospital that serves five governorates in eastern Egypt) | 41.40% (73/176) *K. pneumoniae,* 90.4% (66/73) MDR, 86.3% (63/73) ESBL, and 73.1% (38/52) CR. |
| El-Domany *et al.* 2021^39^ | Jul.2015:  Apr. 2016 | 1005 isolates | Blood, sputum, urine, and wound | Four Hospitals in Kafr Elshaikh | 22.89% (230/ 1005) *KP* distributed as: urine 42.6%, blood 29.13%, sputum 23.9%, and wound 4.3% |
| Sherif *et al.* 2021^40^ | Jul.2018:  Oct. 2018 | 39 MDR *CRKP*  *isolates* | Sputum, BALs, blood, urine, and wound swab | Nein Collaborating Hospitals: National Cancer Institute, Cairo, Alexandria University Research Center, Greater Cairo Tertiary Hospital, and Outpatient Laboratory; Assiut-, Banha-, Mansoura-, Benisweef-, Fayoum-, and Sohag-University Hospitals. | 97.3% MDR *K. pneumoniae* (37/39) distributed in: blood culture 43.6% (17/ 39), respiratory 25.6% (10/39), wound15.4% (6/39), and UTI 15.4% (6/ 39) |
| Azab *et al*. 2021^155^ | April 2015: July 2016 | 292 isolates | Sputum, blood, urine, urethral-,abscess-, nasal-, throat-, vaginal-, ear-, and endo-trachealtube-swaps | Different isolates Egypt, Saudi Arabia , and Sudan | 17% (16/90) *K. pneumoniae* in Egyptian samples versus 14.8% (24/162), 17.5 % (7/40) samples from Saudi Arabia and Sudan, respectively |
| Raheel *et al.* 2020^57^ | Nov. 2016:  Dec. 2018 | 155  isolates | Isolates from patients in the urology, internal medicine, surgical wards, and ICU | Suez Canal University Hospitals | 45.10% (70/155) *Enterobacteriaceae* including*: K. pneumoniae* |
| El-Kholy *et al*. 2020^25^ | 2015: 2016 | 495 isolates | MDR GNP isolates of LRT specimen, pus, urine, and abdominal fluid | Ain-Shams University-, Ain-Shams Specialized University-, and Dar Al-Fouad Hospitals. | 52.7% (266/495) MDR *KP*, included 45.3% CR and 96.2% ESBL (48/495); cIAI 52.2% (91/181), cUTI 43% (80/186), LRTI 71.1% (91/128) |
| Khairy *et al*. 2020^18^ | Mar. 2017:   June 2017. | 250 MDR HAP patients | Sputum, urine, and wound swabs from ICU and orthopedic wards, ICU, medical, surgical-, orthopedic-, and urinary- wards | Minia University Hospital, and Minia Directorate Hospital | 16.8% (42/250) *KP*, (16.8% MDR KP) 100% (42/42) MDR *KP,* distributed in urine 18.1% (16/88), wound swabs 17.3% (13/75), sputum 14.9% (13/87), ICU 41.9% (13/31), orthopedic wards 6.25% (2/32) |
| Hassuna *et al*. 2020^42^ | Feb.2019:  Sep. 2019 | 156 isolates | blood samples-neonatal ICU (sepsis suspected) | Minia University Hospital Neonates ICU | 15.30% (24/156) *KP,* 17% (4/24) MDR, and 83.3% (20/24) XDR |

***CR= carbapenems-resistant; KP= Klebsiella pneumoniae; ETAs= Endotracheal aspirates; VAP= ventilator associated pneumonia; MDR= multi-drug resistant; BAL= Broncho-Alveolary Lavages; ICU= Intensive care unit; GN= Gram-negative; LRTI= lower respiratory tract infection; cIAI= complicated intra-abdominal infections; cUTI= complicated urinary tract infection; UTI= urinary tract infection; HAP= hospital acquired pneumoniae; XDR= extended drug resistance.

***Table 3: Phenotypic prevalence of antimicrobial-resistant Klebsiella pneumoniae isolates in recent Egyptian studies***

| **Site and reference** | **Mabaret El Asafra Labs in Alexandria**^41^ | **Zagazig University Hospital**^72^ | **Four Hospitals in Kafr El-shaikh**^39^ | **Private labs in Egypt, Saudi Arabia & Sudan**^155^ | **Dar Al-Fouad, Ain Shams University, and Specialized Hospitals**^25^ | **Minia Uni., &Directorate Hospitals**^18^ | **Neonatal ICU Al Minia University Hospital**^42^ |
| --- | --- | --- | --- | --- | --- | --- | --- |
| **Method:** | VITEK2®,  Kirby-Bauer test, colistin disk elution, and broth micro-dilution | Modified Kirby– Bauer disk diffusion, colistin broth disk elution, and eCIM | Disk diffusion, VITEK2®, and EDTA combined disc synergy | Kirby–Bauer disc diffusion | Colistin broth microdilution and other methods (not detailed) | Antimicrobial discs and broth microdilution | VITEK2® |
| **P:**  **PEN**  **AMP** | 100% (94/94) | 97.3% (71/73)  30% (71/73) | 98.3%(226/230) | 81.3% (13/16) |  |  | 100% (24/24) |
| **BL/BLI:**  **Pip/Toz**  **Amp/Sulb**  **Amo/Clav** | 100% (94/94) | 97.3% (71/73)  97.3% (71/73)  97.3% (71/73) |  |  | 81.4% (216/266)  94.4% (251/266) |  | 95.8% (23/24)  100% (24/24) |
| **AminoGL:**  **AK**  **Gen** | 63.8: 95.7% | 80.8% (59/73)  85% (62/73) | 70% (161/230) | 62.5% (10/16) | 27.3% (72/266) | 71.4% (30/42) |  |
| **C:**  **CLN/1G**  **FOX/2G**  **CTZ/3G**  **CTX/3G**  **FEP/4G** | 100% (94/94) | 85% (62/73)  85% (62/73)  85% (62/73) |  | 80% (13/16)  81.3% (13/16)  75% (12/16) | 83.3% (221/266)  83.3% (221/266)  99.3% (264/266)  94.4% (251/266) | 90.5% (40/42) | 100% (24/24)  95.8% (23/24)  100% (24/24)  100% (24/24) |
| **Q:**  **CIPR**  **LEV** | 63.8: 95.7%  63.8: 95.7% | 85% (62/73)  85% (62/73) | 76% (175/230) | 81.3% (13/16) | 88% (234/266) | 83.3% (35/42) | 0% |
| **TC:**  **DOX** | 63.8: 95.7% | 11% (8/73) |  | 33.3% (5/16) |  |  |  |
| **CPH** |  | 20.5% (15/73) |  |  |  |  |  |
| **TMP/SMX** | 63.8: 95.7% |  | 60% (138/230) |  |  |  |  |
| **CARB:**  **IMP**  **ETP**  **MEP**  **DOP** | 100% (94/94)  96.9% (/94)  100% (94/94)  78.8% (/94) | 68.5% (50/73)  43.8% (30/73)  71.2% (52/73)  41.1% (30/73) | 21.7% (50/230)  21.3% (49/230)  21.7% (50/230) | 56.3% (9/16) | 36% (96/266)  30.7% (82/266) | 31% (13/42)  57% (24/42) | 95.8% (23/24) |
| **COL** | 27.8% (/94) | 11% (8/73) |  |  | 7.3% (19/266) |  |  |
| **AZT** | 100% (94/94) | 83.6% (61/73) |  |  |  |  |  |

*** Only one method test was performed according to the European Committee on Antimicrobial Susceptibility Testing (EUCAST)^25^, while all other tests were performed according to Clinical and Laboratory Standards Institute (CLSI) guidelines.

***P= penicillins class ; PEN= Penicillin; Amp= Ampicillin; BL/BLI= β-lactam/ β-lactamase inhibitors; Pip/ Toz= piperacillin/tazobactam; Amp/ Sulb= Ampicillin/sulbactam; Amo/Clav= amoxicillin/clavulanic; AminoGL= aminoglycosides; AK= Amikacin; Gen= Gentamicin; C= cephalosporins; CLN= Cefazoline; 1G= first generation; FOX= Cefoxitin; 2G= second generation; CTZ= Ceftazidime, 3G= third generation: CTX= Ceftriaxone; FEP= Cefepime; 4G= 4 generation; Q= Quinolines; CIPR= Ciprofloxacin; LEV= Levofloxacin; TC= tetracycline class; DOX= Doxycycline; CPH= Chloramphenicol; TMP/SMX =Trimethoprim/Sulfamethoxazole; CARB= Carbapenams; IMP= Imipenem, ETP= Ertapenem, MEP= Meropenem; DOP= Doripenem; COL= Colistin; AZT= Aztreonam; eCIM= EDTA-modified carbapenem inactivation method.

**Table 4:** **Genotypic prevalence of antimicrobial resistance genes in Klebsiella pneumoniae isolates in recent Egyptian studies**

| **Site and**  **reference** | | **Mabaret El Asafra Labs, Alexandria** ^41^ | **Zagazig University Hospital**^72^ | **Four Hospitals in Kafr Elshaikh**^39^ | **Nein Collaborating -teaching Hospitals^40^** | **Suez Canal University Hospitals**^57^ | **Dar Al-Fouad, Ain-Shams University & Specialized Hospitals**^25^ | **Minia University, Directorate Hospitals**^18^ | **Neonatal ICU -Al Minia University Hospital**^42^ |
| --- | --- | --- | --- | --- | --- | --- | --- | --- | --- |
| Method | | 2 multiplex PCR | 2 multiplex PCR | PCR | WGS | Conv. PCR | Microarray | PCR | Conv. PCR |
| **CR Genes:** | *bla_NDM_*  *bla_OXA_*  *bla_KPC_*  *bla_VIM_*  *bla_IMP_*  *bla_GES_* | *_NDM_* 92.5%  (87/94)  _OXA-48_ 51.1% (48/94)  *_KPC_* 1.06%  (1/ 94)  bla_VIM_ 0%  bla_IMP_ 0%  bla_GES_ 0% | *_NDM_* 50% (26/52)  _OXA48_ 36.5%  (19/52)  *_KPC_* 11.5%  (6/52)  bla_VIM_ 0%  bla_IMP_ 0% | _NDM-1_ 70% (35/50)  _OXA-48_ 52% (26/50) | _NDM-1_ 3.3%  (13/39)  _NDM-5_ 2.6% (1/39)  _OXA-48_ 30.8%  (12/39)  _OXA-181_ 5.13%  (2/39)  _KPC2_ 2.6%  (1/39) | _NDM_ 88.7% (47/53)  _OXA-48_ 96.2% (51/53)  *_KPC_* 7.5% (4/53)  b_VIM_ 52.8% (28/53) | _NDM-1_ 23.7% (63/266)  _NDM-4_ 0.4% (1/266)  _NDM-5_ 0.3% (8/266)  _OXA-48_ 40.6% (108/266)  _OXA181_ 1.1%  _blaOXA-232_ 4.5% (12/266)  _OXA-244_ 0%  _KPC-2_ 0.8% (2/266) | _OXA48_ 37.5%  (9/24 CR)  _KPC_ 0%  _VIM-1_ 25% (6/24 CR) | _NDM_ 100% (24/24)  oxa-1 75% (18/24)  _KPC_ 95.8% (23/24)  _VIM_ 100% (24/24)  _IMP_ 0%  _SPM_ 0% |
| **AmpC ESBL** | *CTX-M15*  *CTX-M14*  *CTX-M-27*  *CTX-M-55*  *TEM-OSBL*  *TEM-ESΒL*  *SHV-OSBL*  *SHV-12* |  |  |  | 69.2% (27/39) |  | 71.4%  (190/266)  19.5%  (52/266)  4.5%  (12/266)  0%  48.5%  (129/266)  0.4%  (1/266)  51.1%  (136/266)  12.8%  (34/266) |  | _CTX-M_ 100%  (24/24)  SHV 0% |
| **QR**. | *QnrS*  *QnrB*  *QnrA* |  |  |  |  |  |  | 100%  (35/35)  28.5%  (10/35)  0% | 100%  (24/24)  37.5%  (9/24)  0% |

*CR= carbapenem resistance; NDM= New Delhi metallo-β-lactamase; OXA-48= oxacillinase-48; VIM= Verona integron encoded metallo-β-lactamase; IMP= imipenemase metallo-β-lactamase; ESBL= extended spectrum β- lactam; SHV= Sulfhydryl-variable; QR= quinolone resistant; QnrS, qnrB, and qnrA= Plasmid-mediated quinolone resistance (PMQRs) genes; PCR= polymerase chain reaction; WGS= whole-genome sequencing.

***Table 5: Genotypic analysis of Klebsiella pneumoniae virulence genes in recent Egyptian studies***

| **Site & reference** | **Mabaret El Asafra Labs in Alexandria**^41^ | **Nein collaborating Egyptian teaching Hospitals**^40^ |
| --- | --- | --- |
| **Method:** | Polymerase chain reaction (PCR) using Four multiplex PCR kits | Whole-Genome Sequencing (WGS) |
| **Genes:** | Hyper-mucoviscosity 14.9%, each entB, ycfM, mrkD, and fimH 100% (94/94), ybtS 98.9% (93/94), Uge 64.9% (61/94), iutA 58.5% (55/94), rmpA 43.6% (41 /94), Kpn 28.7% (27/94), Each magA, and k2A 0% | Yersiniabactin 69.2% (27/ 39), Hyper-mucoviscosity 15.4% (6/39), Aerobactin 41% (16/39) |

*EntB= enterobactin siderophore system; ycfM= outer membrane lipoprotein; mrkD= type 3 fimbrial adhesion; fimH = type 1 fimbriae; ybtS= yersiniabactin siderophore system; uge= uridine diphosphate galacturonate 4-epimeras; iutA= aerobactin siderophore system; rmpA= the regulator of mucoid phenotype A; Kpn= like fimbrial adhesion; magA= mucoviscosity-associated gene A; and k2A= K2 capsule serotype.

**Table 6: Recent studies on Pseudomonas aeruginosa isolates from Egyptian hospitals**

| **Reference** | **Collection time** | **Sample size** | **Sample type** | | **Sample site** | | | **Prevalence** | | | |
| --- | --- | --- | --- | --- | --- | --- | --- | --- | --- | --- | --- |
| Abdelaziz *et al*. 2024^17^ | Sep. 2019:  Mar. 2020 | 125 of HAI patients | Burns, wounds, urine, sputum, blood, and pus. | | Three different large central hospitalsin Cairoand Mansoura. | | | 56% (70/125) *PA*, 75.7% (53/70) XDR, and 18.5% (13/70) MDR. | | | |
| Edward *et al*. 2024^103^ | Sep.: Nov. 2017 & Jun.: Oct. 2018 | 104 *isolates* | Pus, bronchial lavage, urine, sputum, and blood cultures | | Alexandria Main University Hospital | | | 100% (104/104) *PA* 81.7% (85/104) ESBL PA. | | | |
| Marey et al 2023^102^ | Mar. 2021: Mar. 2023 | 60 VAP  patients | BAL fluid and endotracheal aspirates | | Tanta University Hospitals (ICUs) | | | 25% (15/50) *PA* | | | |
| Azab *et al.* 2021^155^ | April 2015:  July 2016 | 292 isolates | Abscess, blood, urine, ear swab, nasal Swab, endotracheal tube,throat swab, urethral, sputum, and vaginal swab | | | Different private labs in Egypt, Saudi Arabia, and Sudan | | *PA* distributed in Egypt 26.6% (24/90) versus, Saudi Arabia 25.9% (42/162), Sudan 42.5% (17/40). | | | |
| Hassuna *et al.* 2020^100^ | 15 months in 2017& 2019 | 173 isolates | Collected from RTI | | ICUs in Minia University Hospital | | | | | | 100% (137/137) *PA*, 22.5% (39/173) MDR *PA* |
| Bash *et al.* 2020^101^ | May: Sep. 2017 | 100 *isolates* | NA | | | | Kasr El Aini Hospital, & El Borg Labs in Giza | | | 100% (100/100) *PA,* included 59 (59/100) CR, XDR 47% (47/100 *PA*) 79.66% (47/59 CR), MDR 21% (21/100 *PA*) 20.33% (10/5 CR) | |
| Farhan *et al.* 2019^99^ | Oct. 2016  & Feb. 2018 | 530 isolates | Hospitalized patients’ urine, burn swabs, stool, and sputum samples (ICU). | Three Minia hospitals  (Chest, University, and General hospitals) | | | | | 28.3% (150/530) *PA,* 66.6% (100/150) MDR *PA*, 54% (54/100) ESBL *PA*, 21% (21/100) CR *PA,* 52.3% (11/21) MBL | | |

*HAI= hospital acquired infection; PA= Pseudomonas aeruginosa; VAP= ventilator associated pneumonia; BAL=*Bronchoalveolar lavage*; RTI= respiratory tract infections; NA= not applicable; ICU= intensive care unit; XDR= extended drug resistance; MDR= multi-drug resistant; ESBL= extended spectrum beta lactam; CR= carbapenem resistant; MBL= metallo-β-lactamase producers.

***Table 7: Phenotypic prevalence of antimicrobial-resistant Pseudomonas aeruginosa strains in recent Egyptian studies***

| **Site and reference** | **Three central hospitals in Cairo and Mansoura**^17^ | **Alexandria Main University Hospital**^103^ | **Private labs in Egypt, Saudi Arabia & Sudan**^155^ | **Kasr El Aini Hospital, El Borg Labs, Giza**^101^ | **Minia hospitals (high-rate chest hospital)**^99^ |
| --- | --- | --- | --- | --- | --- |
| **Method:** | Kirby-Bauer disk diffusion, Broth microdilution, and MHT | Agar dilution and broth microdilution techniques | Kirby–Bauer disc diffusion | Kirby- Bauer disk diffusion and broth microdilution | Kurby–Bauer disk diffusion |
| **P:**  PIPR  CARPEN |  |  | 100% (90/90 PA) | 64% (64/100 PA)  ,93.2% (55/59 CR) | 46.3% (25/54 ESBL PA), 72.7% (8/11 MBL) |
| **BL/BLI:**  Pip/ Toz  Amo/Clav |  | 38.5% (40/104 PA) |  | 55% (55/100 PA)  ,84.7% (50/59 CR PA) | 100% (54/54 ESBL PA) ,(11/11 MBL) |
| **AmGL:**  AK  Gen | 94.29% (66/70 PA) | 82.7% (86/104 PA) | 41.7% (37/90 PA) | 66% (66/100 PA)  ,91.5% (54/59 CR PA)  70% (70/100 PA)  ,94.9% (56/59 CR PA) | 46.3% (25/54 ESBL PA), 63.6% (7/11 MBL PA) |
| **C:**  CLN/ 1G  FOX/ 2G  CTZ/ 3G  CTX/ 3G  FEP/ 4G |  | 67.3% (70/104 PA)  82.7% (86/104 PA) | 61.5% (55/90 PA)  79.2% (71/90 PA)  66.7% (66/90 PA) | 53% (53/100 PA)  ,83.08% (49/59 CR AP)  74% (/100 PA)  ,100% (59/59 CR PA) | 100% (54/54 ESBL PA)  ,72.7% (8/11 MBL PA) |
| **Q:**  CIPR  LEV  MOX | 92.86% (65/70 PA)  92.86% (65/70 PA) | 85.6% (89/104 PA)  83.7% (87/104 PA)  83.7% (87/104 PA) | 47.8% (43/90 PA) | 64% (64/100 PA),  ,94.9% (56/59 CR PA)  67% (67/100 PA)  ,100% (59/59 CR PA) | 35.2% (/54 ESBL PA)  ,36.4% (11 MBL PA)  55.6% (/54 ESBL PA)  36.4% (/11 MBL PA) |
| **TC:**  TIG |  |  | 83.3% (75/90 PA) |  | 42.6% (23/54 ESBL PA), 45.5 (5/11 MBL PA) |
| SUL |  |  | 78.3% (70/90 PA) |  |  |
| **CARB:**  IMP  MEP | 55.71% (39/70 PA) | 86.5% (90/104 PA)  76% (79/104 PA) | 25% (23/90 PA) | 100% (59/59 CR PA)  96.6% (57/59 CR PA) |  |
| AZN (Mon)  PolyMxB  COL | 24.29% (17/70 PA)  95.71% (67/70 PA)  92.86% (65/70 PA) | 5.8% (6/104 PA) |  | 23% (23/100 PA)  ,27.1% (16/59 CR PA) | 83.3% (/54 ESBL PA)  ,54.5% (/11 MBL PA)  74.1% (/54 ESBL PA)  ,54.5% (/11 MBL PA)  37% (/54 ESBL PA)  ,36.4% (/11 MBL PA) |
| **MACRO** |  |  | 100% (90/90 PA) |  |  |

*ِAll tests were performed according to CLSI guidelines.

*P= penicillins class ; PIPR= Pipracillin; CARPEN= carbenicillin; BL/BLI= β-lactam/ β-lactamase inhibitors; Pip/ Toz= piperacillin/tazobactam; Amo/Clav= amoxicillin/clavulanic; AmGL= aminoglycosides; AK= Amikacin; Gen= Gentamicin; C= cephalosporins; CLN= Cefazoline; 1G= first generation; FOX= Cefoxitin; 2G= second generation; CTZ= Ceftazidime, 3G= third generation; CTX= Ceftriaxone; FEP= Cefepime; 4G= 4 generation; Q= Quinolines; CIPR= Ciprofloxacin; LEV= Levofloxacin; MOX= Moxifoxacin; TC= tetracycline class; TIG= Tigecycline; SUL= sulfonamide; CARB= Carbapenams; IMP= Imipenem; MEP= Meropenem; AZN= Azitronam; MON= monobactam; PolyMxB= Polymyxin B; COL= Colistin; MACRO= macrolide; MHT= modified Hodge test; *PA= Pseudomonas aeruginosa;* CR= carbapenem resistant; ESBL= extended spectrum β-lactama; MBL= mettalo-β- lactam.

***Table 8: Genotypic prevalence of antimicrobial resistance genes in Pseudomonas aeruginosa isolates in recent Egyptian studies***

| **Site and**  **reference** | | **3 Central hospitals in Cairo & Mansoura** ^17^ | **Alexandria Main University Hospital** ^103^ | **Kasr El Aini Hospital, &El Borg Labs in Giza** ^101^ | | **Minia Chest, University, and General hospitals** ^99^ | |
| --- | --- | --- | --- | --- | --- | --- | --- |
| Method | | PCR and molecular typing | PCR and plasmid profiling | | PCR | | PCR |
| **CR** | *bla_NDM_*  *bla_OXA_*  *bla_PER_*  *bla_VIM_*  *bla_IMP_*  *bla_GIM_*  *bla_SPM_* | *_NDM-1_*:74.3% (52/70)  *_VIM-1_*: 45.7% (32/70)  *_VIM-2_*: 21.4% (15/70) | *_Oxa-10_:* 64.4%(67/104)  1.9% (2/104)  2.9% (3/104) | *_NDM-1_*: 90.9% (10/11)  *_VIM-1_*: 18.1% (2/11)  *_VIM-2_*: 0%  0% | | 52.3% (11/21)  42.8% (9/21)  52.3% (11/21)  38% (8/21) | |
| **ESBL** | *bla_CTX-M15_* |  |  |  | | 55.5% (30/54 ESBL) | |
| **AminoG**. | *aac(6ʹ)-lb*  *aac(3)-II*  *rmtB*  *aph (3’)-I* | 84.30% (59/70)  81.40% (57/70)  74.30% (52/70)  31.40% (22/70) |  |  | |  | |
| **Colistin** | *mcr-1* | 12.9% (9/70). |  |  | |  | |

*PCR= polymerase chain reaction; CR= carbapenem resistance; NDM= New Delhi metallo-β-lactamase; OXA-48= oxacillinase-48; VIM= Verona integron encoded metallo-β-lactamase; and IMP= imipenemase metallo-β-lactamase**;** and ESBL= extended spectrum β-lactama; AminoG.= aminoglycoside resistance genes (included: *aac(6ʹ)-lb, aac(3)-II, rmtB, and aph (3’)-I* ).

**Table 9: Recent studies on Staphylococcus aureus isolates from Egyptian hospitals**

| **Publication** | **Sampling time** | **Sample size** | **Sample type** | **Hospitals** | **Prevalence** |
| --- | --- | --- | --- | --- | --- |
| Esmaeel *et al.* 2025^133^ | Sep 2023: Mar. 2024 | 500 ICU patients | pus aspirate, wound swabs, sputum, BAL, blood, urine, CSF, and conjunctival swabs. | Zagazig University Hospitals | 70% (n= 65) *S. aureus* out of *92 Staphylococci* isolates.  70.3% (n=26) macrolide-resistant *S. aureus,* including 61.5% (16/26) MRSA |
| Monecke *et al.* 2023^16^ | 5 months in 2020 | 30 isolates | Aspirated pus, wound swab, blood culture, bone marrow aspirate, catheter tip, peritoneal fluid, sputum, throat swab, and urine | Medical Research Institute, Alexandria University | 100% (30/30) *S. aureus* distributed in aspirated pus 60% (18/30), wound swab 13.3% (4/30), blood culture 6.7% (2/30), and 3.3% (1/30) for each bone marrow aspirate, catheter tip, peritoneal fluid, sputum, throat swab, and urine |
| Alfeky *et al.* 2022^105^ | Sep. 2019: Dec. 2018 | 170  *isolates* | Wound swabs, blood, sputum, urine, pus of an abscess, eye swabs, IV catheters, ETAs, and urinary catheters. | Al-Sayed Galal and Al-Demerdash University Hospitals | 100% (170/170) *S. aureus* in: wound swabs 34.1% (58/170), blood 15.3% (26/170), IV catheters 10% (17/170), urine 8.8% (15/170), sputum 10% (17/170), abscess pus 8.8% (15/170), ETAs 5.9% (10/170) eye swabs 5.9% (10/170), and urinary catheters 1.2% (2/170) |
| El-Sweify *et al.* 2021^107^ | 10 months | 474 HAI  patients | skin and soft tissue, blood, CSF, lower respiratory tract, and osteomyelitis | Suez Canal University Hospitals | 25.3% (120/474) *S. aureus* |
| Azab *et al*. 2021^155^ | Apr. 2015: Jul. 2016 | 292 isolates | Abscess, blood, ear swab, endotracheal tube, sputum, nasal swab, throat swab, urethral, urine, and vaginal swab | Different private labs in Egypt, Saudi Arabia, and Sudan | 15.3% (45/292) *S*. *aureus* distributed in: Egypt 18.8% (17/90), versus Saudi Arabia 17.2% (28/162), and in Sudan 0% |
| Ibrahim *et al.* 2020^106^ | Jan 2016: Dec 2017 | 379 Gm+ isolates | Urine, pus, throat swab, blood, seminal fluid, prostatic fluid, sputum swab, ascetic fluid, skin swab, nipple discharge, and urinary catheter | Al-Demerdash University Hospital | 68% (258/379) *S. aureus* distributed in: Urine 40%, pus 33.3%, throat swab 12.8%, blood 3.1%, seminal fluid 3.1%, prostatic fluid 3.1%, sputum swab 1.2%, ascetic fluid 0.8%, skin swab 1.5%, nipple discharge 0.8%, and urinary catheter 0.3%.  70 (180/258) MRSA distributed in pus 57.1%, urine 30%, skin swab 5.71%, throat swab 2.9%, (blood, urinary catheter , sputum swab) 1.43%, and absent in nipple discharge, seminal-, prostatic- and ascetic-fluid |

*S. aureus= Staphylococcus aureus; Gm+= Gram Positive; IV= intravenous catheters, ETAs= endotracheal aspirates; HAI= hospital acquired infection; CSF= cerebrospinal fluid; ICU= intensive care unit; BAL= bronchoalveolar lavage.

***Table 10:*** ***Phenotypic prevalence of antimicrobial-resistant Staphylococcus aureus strains in recent Egyptian studies***

| **Site and reference** | **Medical Research Institute, Alexandria University**^16^ | **Al-Sayed Galal and Al-Demerdash University Hospitals**^105^ | **Suez Canal University Hospitals**^107^ | **Al-Demerdash University Hospital**^106^ |
| --- | --- | --- | --- | --- |
| **Method:** | Disk diffusion | Kirby-Bauer and Cefoxitin disk diffusion/ agar, Oxacillin agar | Disk diffusion, and Cefoxitin disk diffusion | Agar Disc diffusion |
| **P:**  PEN  AMOX |  | 81.2% (138/170 SA), 100% (138/138 MRSA) |  | 100% (70/70 MRSA) |
| **AmGL:**  AK  Gen  Tob | 53.3% (16/30 MRSA)  90% (27/30 MRSA)  90% (27/30 MRSA) | 21.8% (73/170 *SA*),  26.1% (36/138 MRSA)  49.4% (84/170 *SA*), 53.6% (74/138 MRSA) | 0% PVL+MRSA  31.8% (21/66 PVL-MRSA) | 77.1% (54/70 MRSA)  77.1% (54/70 MRSA) |
| **C:**  FOX/ 2G | 100% (30/30 MRSA) | 81.2% (138/170 *SA*), 100% (138/138 MRSA) |  |  |
| **Q:**  CIPR  LEV | 43.3% (13/30 MRSA)  26.7% (8/30 MRSA) | 26.5% (45/170 *SA*),  31.1% (43/138 MRSA)  25.3% (43/170 *SA*),  29.7% (41/138 MRSA) | 25% (6/24 PVL+MRSA)  ,31.8% (21/66 PVL-MRSA) | 74.3% (52/70 MRSA) |
| **TC:**  TIG  DOX | 13.3% (4/30 MRSA)  46.7% (14/30 MRSA) | 47.6% (81/170 *SA*),  56.5% (78/138 MRSA)  48.8% (83/170 *SA*),  57.2% (79/138 MRSA) |  | 71.4% (50/70 MRSA) |
| **CPH** | 23.3% (7/30 MRSA) | 24.1% (41/170 *SA*),  27.5% (38/138 MRSA) |  | 74.3% (52/70 MRSA) |
| TMP/SMX | 56.7% (17/30 MRSA) | 11.2% (19/170 *SA*),  13.8% (/138 MRSA) |  |  |
| **CARB:**  IMP |  |  |  | 78.6% (55/70 MRSA) |
| CM  EM  AZM(Mon)  Rif  VM  CLRM | 33.3% (10/30 MRSA)  40% (12/30 MRSA)  13.3% (4/30 MRSA)  0% (0/30 MRSA) | 29.4% (50/170 *SA*),  36.2% (50/138 MRSA)  37.6% (64/170 *SA*),  46.4% (64/138 MRSA)  27% (46/170 *SA*),  33.3% (46/138 MRSA)  12.4% (21/170 *SA*),  15.2% (21/138 MRSA) | 29.1% (7/24 PVL+MRSA)  ,37.8% (25/66 PVL-MRSA)  25% (6/24 PVL+ MRSA), 45.4% (30/66 PVL-MRSA)  0% PVL+/-MRSA | 62.8% (44/70 MRSA)  78.6% (55/70 MRSA)  50% (35/70 MRSA)  78.6% (55/70 MRSA) |

*** Only one method test was performed according to the EUCAST guidelines^106^, while all other tests were performed according to CLSI guidelines.

*P= penicillins class ; PEN= Penicillin; AMOX= amoxicillin; AmGL= aminoglycosides; AK= Amikacin; Gen= Gentamicin; Tob= Tobramycin; C= cephalosporins; FOX= Cefoxitin; 2G= second generation; Q= Quinolines; CIPR= Ciprofloxacin; LEV= Levofloxacin; TC= tetracycline class; TIG= Tigecycline; DOX= Doxycycline; CPH= Chloramphenicol; TMP/SMX =Trimethoprim/Sulfamethoxazole; CARB= Carbapenams; IMP= Imipenem; CM= Clindamycin; EM= Erythromycine; AZM= Azithromycine; Mon= Monobactams; Rif= Rifampin; VM= vancomycin; CLRM= Clarithromycin; TP= teicoplanin; LZ= Linezolid; ; *SA*= *Staphylococcus aureus; MRSA*= methicillin resistant *Staphylococcus aureus.*

**Table 11: Genotypic prevalence of antimicrobial resistance genes in Staphylococcus aureus isolates in recent Egyptian studies**

| **Site and**  **reference** | | **Zagazig University Hospitals**^133^ | **Medical Research Institute, Alexandria University**^16^ | **Al-Sayed Galal and Al-Demerdash University Hospitals**^105^ | **Suez Canal University Hospitals**^107^ |
| --- | --- | --- | --- | --- | --- |
| Method | | PCR | Multiplex PCR and Array | Conv. PCR | PCR |
| **MRSA** | *mecA*  *femA* |  | 97% (33/34)  91% (31/34) | 81.2% (138/170) | 75% (90/120) |
|  | *PVL* |  | 40% (12/30) |  | 26.7% (24/90) |
| **MACRO** | *ermA*  *ermB*  *ermC*  *msrA* | 15.4% (4/26)  29.7% (11/26)  24.3% (9/26) | 3.3% (1/30)  3.3% (1/30)  13.3% (4/30)  16.7% (5/30) |  |  |

**MRSA*= methicillin-resistant *Staphylococcus aureus*; and MACRO= macrolide resistance genes.
